# Advantages of Bimetallic Organic Frameworks in the Adsorption, Catalysis and Detection for Water Contaminants

**DOI:** 10.3390/nano13152194

**Published:** 2023-07-28

**Authors:** Jun Luo, Xiao Luo, Yonghai Gan, Xiaoming Xu, Bin Xu, Zhuang Liu, Chengcheng Ding, Yibin Cui, Cheng Sun

**Affiliations:** 1 Nanjing Institute of Environmental Sciences, Ministry of Ecology and Environment of the People’s Republic of China, Nanjing 210042, China; luojun@nies.org (J.L.);; 2 Department of Chemistry, Tsinghua University, Beijing 100084, China; 3 State Key Laboratory of Pollution Control and Resource Reuse, School of the Environment, Nanjing University, Nanjing 210023, China

**Keywords:** bimetallic organic framework, adsorption, catalysis, detection, water environment

## Abstract

The binary metal organic framework (MOF) is composed of two heterometallic ions bonded to an organic ligand. Compared with monometallic MOFs, bimetallic MOFs have greatly improved in terms of structure, porosity, active site, adsorption, selectivity, and stability, which has attracted wide attention. At present, many effective strategies have been designed for the synthesis of bimetallic MOF-based nanomaterials with specific morphology, structure, and function. The results show that bimetallic MOF-based nanocomposites could achieve multiple synergistic effects, which will greatly improve their research in the fields of adsorption, catalysis, energy storage, sensing, and so on. In this review, the main preparation methods of bimetallic MOFs-based materials are summarized, with emphasis on their applications in adsorption, catalysis, and detection of target pollutants in water environments, and perspectives on the future development of bimetallic MOFs-based nanomaterials in the field of water are presented.

## 1. Introduction

Since the concept of metal organic framework (MOF) was formally proposed in 1995 [1], the unique material has received extensive attention. Metal–ligand compounds, such as materials of the Institute Lavoisier (MIL) [2,3] and zeolitic imidazolate frameworks (ZIFs) [4], formed by bonding of transition metals and organic ligands, possess all kinds of unique structural properties, including but not limited to large specific surface area, developed pore structure, physical and chemical relative stability, and adjustable structure and function [5,6].

These MOFs, which contain only one transition metal, are favored by researchers, who promote the in-depth development of MOFs in energy, materials, sensors, the environment [7,8,9,10], and other fields for application. Li et al. [11] used a household microwave oven to successfully synthesize Zr_6_O_4_(OH)_4_(BTC)_2_(HCOO)_6_ (MOF-808) nanoparticles with a maximum adsorption capacity of 24.83 mg/g for arsenic solution. Singh et al. [12] prepared the g-C_3_N_4_/Cu-DTO MOF nanocomposite with heterojunction structure by ultrasound to combine g-C_3_N_4_ and Cu-DTO MOF in methanol solution. At room temperature, the lowest detection limit of the material for endocrine disruptors could be acquired at 0.02 µM. Nguyen et al. [13] used MIL-101(Fe) and g-C_3_N_4_ as precursors and adopted solvothermal synthesis, synthesizing the MOF derivative nanomaterial of a three-dimensional g-C_3_N_4_/MIL-101(Fe). The presented material showed excellent performance in the photocatalytic degradation of paracetamol. For more application cases of monometallic MOFs, Gonzalez et al. [14] carried out a detailed review.

However, as researchers pursued higher properties of MOF materials, these monometallic MOFs and their derivatives were no longer able to further meet the needs, which gradually led to the design and development of binary metal organic frameworks with higher properties [15,16]. At present, in the field of water environmental treatment, binary metal MOFs and their derivatives (the bimetallic MOF-based nanomaterials) [17,18] have been greatly developed compared with monometallic MOFs. Typically, Yaqoob et al. [19] designed the Fe-Ni-MOF/nanotube composite catalysts using conventional hydrothermal reaction processes, which significantly improved the redox reaction of Fe-MOF or Ni-MOF. Bathla et al. [20] reported multiple bimetallic nanostructure metal-organic frameworks to photodegrade harmful organic compounds. The report confirmed the significant role of bimetallic MOFs in the photocatalytic degradation of organic pollutants.

In recent years, bimetallic MOF derivatives have been directly prepared to form a variety of MOF-based nanomaterials. Research on a bimetallic magnetic nanocomposite, Fe_3_O_4_@CeO_2_@MOF-5(Zn-Co), to adsorb flusilazole in a water environment was noteworthy. The specific surface area of the bimetallic MOFs-based nanomaterial could reach 399 m^2^/g, which was more than 10 times that of a single metallic Fe_3_O_4_@CeO_2_@MOF-5(Zn) or Fe_3_O_4_@CeO_2_@MOF-5(Co) [21]. The report of vanadium/chromium-bimetallic MOFs in adsorption desulfurization showed a pleasing advantage. A special interaction between the bimetal and sulphur atoms greatly promoted the van der Waals force between the material and the target pollutants [22], thus improving the selectivity of the material for thiophene and aromatics. Selvam et al. [23] achieved encouraging results in core-shell CuNi bimetallic nanoparticle electrochemical biosensors, which made great progress in detecting dopamine.

Bimetallic MOF-based composites have many advantages over monometallic MOF-based materials: (i) Different monometallic MOFs have different metallic elements, organic ligands, morphology, and structure, which can be prepared by different combination strategies to construct bimetallic MOF materials with different compositions, structures, and functions; (ii) Most monometallic MOFs have undeveloped holes, but the synthesis of binary metal MOFs can further effectively improve this property. In addition, the preparation process of bimetallic MOFs is relatively simple and mild; (iii) The regular staggered arrangement of metal ions (or metal ion clusters) and organic ligands in bimetallic MOF structures is conducive to further fixing and dispersing metals (or metal oxides), improving the stability and catalytic activity of materials.

Based on the advantages mentioned above, many studies have reported binary metal MOF-based nanomaterials with different compositions and structural properties that are widely used in environmental pollution control [24,25,26]. Fan et al. [27] focused on the synthesis of bimetallic MOFs and catalytic degradation of water pollutants and further summarized in detail the influencing factors and catalytic mechanism of this material in the activation processes for peroxydisulfate (PDS), peroxymonosulfate (PMS), and peroxide (H_2_O_2_). Soni et al. [24] reported the latest developments in the synthesis and electrocatalytic activity of two-dimensional MOFs with bimetallic nodes. Kumari et al. [28] summarized the application of MOF-derived materials for H_2_ and CO_2_ adsorption and photocatalysis. However, the application of bimetallic MOF-based materials in the environment is not detailed in their reviews. Sanati et al. [29] concentrated on the superior performance of bimetallic materials derived from metal-organic frameworks in electrochemical energy storage, while Chen et al. [30] focused on the design, construction, and synthesis of bimetallic MOF-based materials. Differently, we mainly aim at the bimetallic MOFs-based nanomaterials in the remediation of water environments and summarize the applications of the materials in adsorption, catalytic degradation, and detection of pollutants.

**Figure 1:**
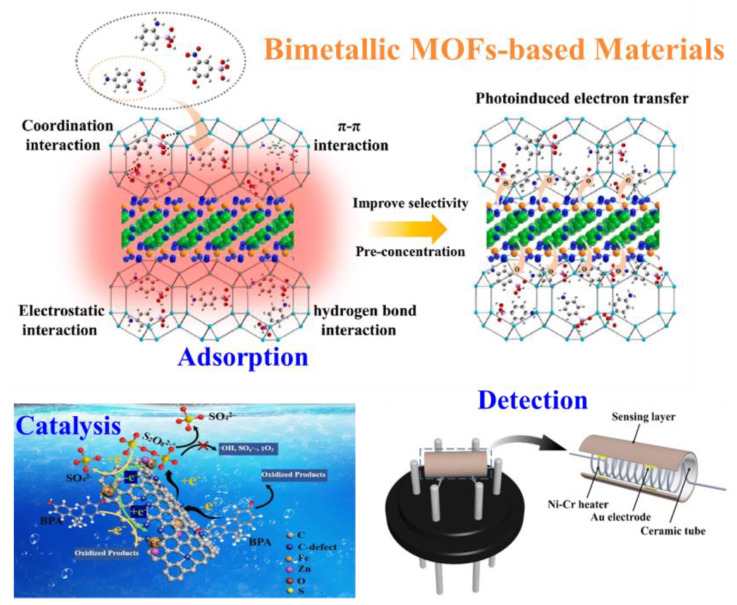
Classification and applications for bimetallic MOF-based nanomaterials. S_2_O_8_^2−^* represents the intermediate formed after S_2_O_8_^2−^ has acquired electrons. Reproduced with permission from ref. [33,36,42]. Copyright 2022, Elsevier.

This review provides the important progress of bimetallic MOFs in the treatment of environmental pollutants in water [31,32,33]. The synthesis process of bimetallic MOFs is summarized. The application of bimetallic MOFs in the adsorption, catalysis, and detection of pollutants in water was emphasized. Finally, the future development and design of bimetallic MOF-based nanomaterials in the field of water environment are prospected. By discussing and examining previous studies, we hope to provide some ideas on the design and construction of bimetallic MOFs-based nanomaterials for effective treatment of water environmental pollutants [34,35,36,37,38,39,40,41,42], so as to stimulate more applications of bimetallic MOFs-based nanocomposites in the environmental field (Figure 1).

## 2. Preparation Strategies of Bimetallic MOF-Based Nanocomposites

As is well known, the preparation methods and synthesis processes of materials are of great importance to their morphology, structure, and performance characteristics. According to statistics, the types of MOFs formed by the bonding of many organic ligands with metal ions have already exceeded 2000 [43]. Mainstream methods for synthesizing these monometallic MOF materials have been summarized in detail in the report of Mukherjee and co-workers [44], including hydrothermal, microwave irradiation, the ionic liquid method, and so on. These basic strategies for the synthesis of monometallic MOF materials have also been adopted by researchers for the construction and development of bimetallic MOF-based materials. In addition, binary metal MOF-based composites are also further extended by some major post-treatment methods [30], such as heat treatment, mechanical processing, the sol–gel method, and the like. Thus, the synthesis methods mentioned above for bimetallic MOFs can be divided into two categories. One is direct synthesis, namely hydrothermal, solvothermal, mechanochemistry, electrochemistry, sonochemistry, and spray-drying. Another is indirect synthesis or post-treatment, that is, pyrolysis, ball-milling, metal-organic chemical vapor deposition, and solution impregnation.

### 2.1. Direct Synthesis

Here, the direct synthesis of binary metal MOFs is the self-assembly process of metal ions with organic ligands to form a coordination polymer in one pot. Bimetallic MOFs prepared by direct synthesis have retained their physical and chemical properties since the beginning of synthesis, including a large specific surface area, a developed pore structure, high conductivity, and a great Fermi level.

The synthesis and exploration of bimetallic MOFs have become important ways to develop new MOF nanomaterials. Liu et al. [45] synthesized 36 kinds of bimetallic MOFs (MnFe-MOFs, MnMg-MOFs, MgCo-MOFs, CoNi-MOFs, FeNi-MOFs, MnNi-MOFs, and so on) using dimethyl formamide (DMF) as solvent by the hydrothermal method. The structural properties of the synthesized MOFs were investigated by UV-vis diffuse reflectance spectroscopy (DRS) and X-ray photoelectron spectroscopy (XPS). The results showed that the structural properties of bimetallic MOFs were better than those of monometallic MOF materials. Chen et al. [46] successfully prepared CoNi bimetallic MOFs (CoNi-MOFs) with a rough surface and spherical shape via a typical one-step solvothermal method (Figure S1a). Galvanostatic charge/discharge (GCD) and cyclic voltammetry (CV) were used to test the electrochemical properties of CoNi-MOFs. The results displayed that the maximum specific capacitance of the structured material could be increased to 2608 F/g at 1 A/g, and it still maintained good stability after 5000 cycles of discharge.

In order to achieve higher structure, morphology, and performance advantages, a variety of binary metal MOF materials with strictly controlled preparation processes were compounded. Kuila and co-researchers had successfully synthesized a structurally stable 3d–4f bimetallic coordination polymer, CeFe MOFs [47]. Specifically, cerium nitrate hexahydrate, iron nitrate hexahydrate, and 2-aminoterephthalic acid were used as raw materials, with DMF, methanol, and water as solvents to prepare the CeFe MOFs. The visible light absorption characterization analysis exhibited that the absorption intensity of CeFe MOFs synthesized was obviously better than that of Fe-MOF and Ce-MOF. The photocatalytic degradation tests further confirmed the excellent performance of electron–hole pair separation in the visible band, and the degradation efficiency of 10 ppm of acetaminophen reached 94.6% in 210 min. Suksatan and colleagues synthesized nanometer nickel-zinc bimetallic MOFs (NiZn-MOFs Figure S1b) with uniform morphology with the ultrasonic method [48]. Fourier transform infrared spectroscopy, specific surface area, and thermogravimetric analysis showed that, compared with monometallic materials (Ni-MOF and Zn-MOF), the samples of NiZn-MOFs had a larger Brunauer–Emmet–Teller (BET) specific surface area and porosity and higher thermal stability.

### 2.2. Indirect Synthesis or Post-Treatment

Indirect synthesis, or post-treatment method, is further developed on the basis of direct synthesis for the product follow-up treatment so as to form the final binary metal MOFs-based material. The end-product is a kind of derivatively bimetallic MOF materials that are markedly different from those formed by direct synthesis in terms of structure, morphology, chemical functional groups, and properties. What needs illustration is that the target product of bimetallic MOF-based materials cannot be achieved by the one-pot synthesis method.

Guo and co-team [49] first synthesized cobalt–molybdenum bimetallic MOFs (CoMo-MI) by solvothermal method and further heated treatment to effectively design the cobalt-molybdenum bimetallic MOF-based carbon and nitrogen nanomaterials (CoMo-MI-T). Figure S2a shows the preparation process for CoMo-MI-T. These results of multiple characterization techniques (XRD, SEM, TG, and TEM) demonstrated that, compared with CoMo-MI, the crystal structure of CoMo-MOFs for CoMo-MI-T nanomaterials had collapsed and the surface morphology was rough. Meanwhile, the CoMo-MI-T’s thermal stability was enhanced, and the content of micropores and mesoporous developed. Electrochemical performance analysis confirmed that the CoMo-MI-T nanomaterials possessed a better synergistic effect among the multiple components, which promoted electron conduction, thus enhancing the electrocatalytic oxygen evolution reaction. Liang and his co-research group [50] explored CoNi-MOF as a precursor to the pyrolysis preparation of CoNi@C nanowires with Janus structures (Figure S2b). The material showed extremely strong stability and sustainable recycling in the long-term heating and cooling cycle tests, which could be used as a high-performance material to prevent electromagnetic interference in low- or high-temperature environments. Analogously, Wang et al. [51] used cobalt-cerium bimetallic MOFs as intermediates to produce the CoCeO_x_ nanocomposites (CoCe-MOF) (Figure S2c). In the experiments of catalytic conversion of carbon dioxide to ethanol, the ethanol conversion efficiency of the CoCe-MOF experimental groups could reach 97%, and almost no acetone byproduct was produced. In the 40 h test period, the ethanol conversion by the CoCe-MOF catalyst was only reduced by 3%, while the Co_3_O_4_/CeO_2_ control groups significantly reduced it by 11%.

**Figure 2:**
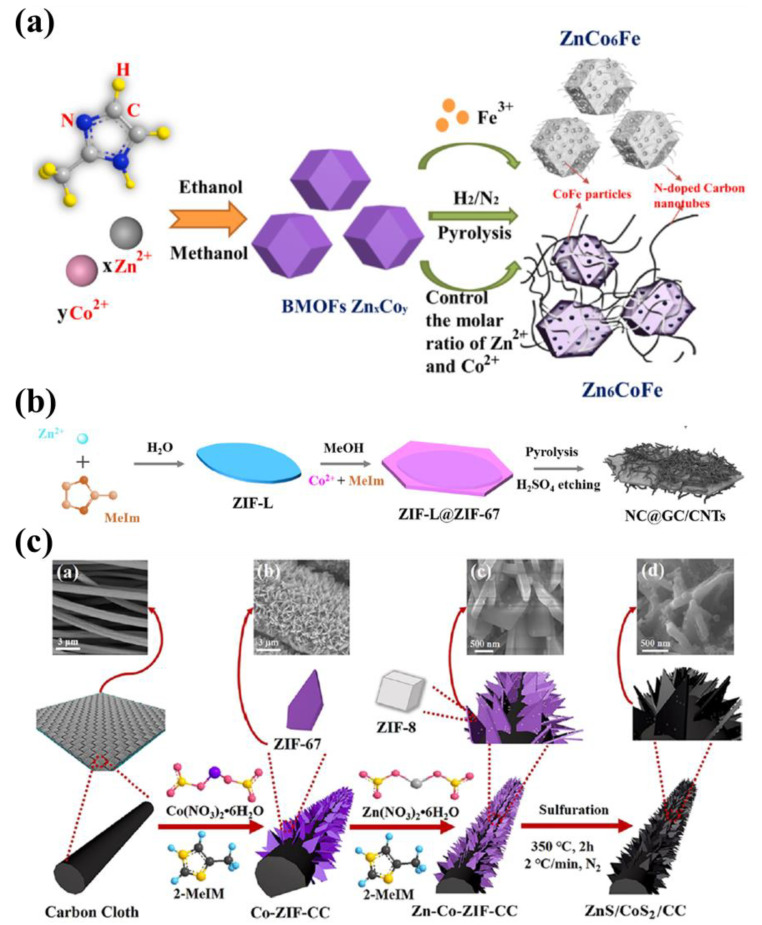
(**a**) Schematic illustration for the formation process of Zn_6_CoFe. Reproduced with permission from ref. [52]. Copyright 2020, Elsevier. (**b**) Leaf-shape core-shell structured ZIF-L@ZIF-67 and its transformation to NC@GC/CNTs. Reproduced with permission from ref. [53]. Copyright 2022, Elsevier. (**c**) Schematic diagram of the synthesis process of ZnS/CoS_2_/CC. Reproduced with permission from ref. [54]. Copyright 2022, American Chemical Society.

In Figure 2a, Yan et al. [52] demonstrated the synthesis of zinc–cobalt bimetallic MOFs (BMOFs Zn_x_Co_y_) with methanol and ethanol as solvents. Then, ferric ions were introduced into BMOF Zn_x_Co_y_ and heated in the gas mixture of H_2_ and N_2_ to prepare an MOF-based Co-Fe bimetallic composite (ZnCo_6_Fe). Scanning electron microscope analysis made clear that BMOF Zn_x_Co_y_ was smooth and regular polyhedral, while ZnCo_6_Fe was composed of ZnCo-MOF derivatives and carbon nano tubes (CNTs) networks with a rough surface. Afterward, the material was used to test the microwave absorption properties, and the minimum microwave absorption reflection loss of ZnCo_6_Fe was −66 dB. The mechanism analysis further proved that the defective structure of the materials could greatly improve the microwave absorption characteristics and provide workable ideas for the design and construction of highly efficient microwave-absorbing materials.

A novel preparation method for bimetallic MOF-based nanocomposites has been successfully brought up by Zhang et al. [53], which mainly included three stages (Figure 2b): Firstly, Zn-MOF (ZIF-L) was synthesized by the hydrothermal method with Zn^2+^ and 2-methylimidazole. Secondly, on the basis of ZIF-L, ZIF-67 was further synthesized to form the product ZIF-L@ZIF-67. Finally, under the conditions of concentrated sulfuric acid and heating, the bimetallic MOFs ZIF-L@ZIF-67 were etched and cracked into bimetallic MOF-based nanocomposites (NC@GC/CNTs) with similar leaf shapes. The highlight of the method was mainly reflected in the pyrolysis stage of MOF-based material synthesis. Through reasonable control of the amount of concentrated sulfuric acid, the physical and chemical properties of the end-products NC@GC/CNTs*_x_* (*x* = 1, 2, 3, and 4) could be effectively regulated. In laboratory conditions, NC@GC/CNTs*_2_* displayed a surprising advantage in the adsorption of NaCl (1000 mg/L sodium chloride aqueous solution), where the maximal adsorption capacity attained 77.33 mg/g after 30 min.

Recently, another interesting pyrolysis method for preparing bimetallic MOF-based nanocomposites was worth noting. Fu et al. [54] cleverly used the stepwise synthesis method to synthesize ZIF-67 and ZnCo-MOFs (ZnCo-ZIF-CC). Subsequently, in the pyrolysis stage, sulfur powder was co-pyrolyzed with ZnCo-ZIF-CC to produce sulfide bimetallic MOFs-based material (ZnS/CoS_2_/CC). The schematic diagram of the preparation process is shown in Figure 2c. The ZnS/CoS_2_/CC was used as anode material for lithium batteries, demonstrating the satisfactory advantages of electric energy storage. The battery capacity of the material was still up to 1644.7 mA h/g after hundreds of cycles of testing at the high current density of 1 A/g.

Furthermore, mechanical grinding or ball-milling has also proven to be an effective strategy for the synthesis of bimetallic MOF-based nanocomposites. Panda et al. [55] synthesized amorphous bimetallic MOF intermediates using ball-milling the precursors (Al-MOF (Al-ndc) and Ga-MOF (Ga-ndc)); afterward, the bimetallic MOF-based nanomaterial AlGa-MOFs with crystal structure were reconstructed via steam treatment. This study highlighted the importance of ball-milling technology in bimetallic MOF preparation and also stressed the limitations of traditional MOF preparation methods in bimetallic MOF synthesis.

The feasible method for preparing bimetallic MOF-based nanomaterials by ball-milling and heat treatment is attracting more and more attention [56,57]. Based on the monometallic MOF (Fe-Tz and Cu-Tz) of iron and copper [56], M. Lee et al. mixed the two by mechanical grinding to prepare a bimetallic MOF intermediate (FeCu-*x*:*y*) with evenly dispersed iron and copper, then pyrolyzed, which finally formed the required bimetallic MOF-based nanomaterials (FeCu-*x*:*y-T*). For comparison, a different preparation process was also performed, which exhibited the transformation from one-pot synthesis of FeCu-MOFs (FeCu-*50*:*50*DEF) by hydrothermal method to the preparation of bimetallic MOF-based nanomaterials (FeCu-*50*:*50-T*DEF) with heat. Morphology and microstructure analysis showed that the distribution of elements in the FeCu-*x*:*y-T* sample was more uniform than that in the FeCu-*50*:*50-T*DEF. The results of the redox reaction confirmed that the synergistic effect of the FeCu-*x*:*y-T* sample was greater than that of the FeCu-*50*:*50-T*DEF, which underlined the effectiveness of mechanical grinding and pyrolysis in bimetallic MOF-based nanomaterials.

## 3. Applications of Bimetallic MOFs-Based Nanocomposites for Pollutants in Water

Bimetallic MOF-based nanocomposites have been widely regarded as novel functional materials for controlling pollutants in environmental water bodies due to their advantages in structure and performance. Therefore, many new bimetallic MOFs with different forms have been developed and constructed, which have been applied in adsorption removal, catalytic degradation, and the detection of water pollutants.

### 3.1. Adsorption

Bimetallic MOF-based nanomaterials are characterized by a larger surface area, a richer pore structure, and more available metal sites than monometallic MOF-based materials. Therefore, it has important practical significance in developing porous bimetallic MOF-based materials to absorb ions or molecules in water to a greater extent. At the same time, reasonable and effective adjustment of the composition of bimetallic MOFs-based materials, including metal and ligand types, can further improve the adsorption strength of the materials on target ions or molecules in water. By referring to existing reports, it can be found that the adsorption of bimetallic MOF-based nanocomposites on ions or molecules is highly correlated with the structure of MOFs [58,59,60,61,62,63,64,65,66]. Consequently, part of the latest research is summarized about the adsorption–removal of pollutants in water environments by bimetallic MOF-based materials in Table 1.

#### 3.1.1. Ionic Adsorption

**Figure 3:**
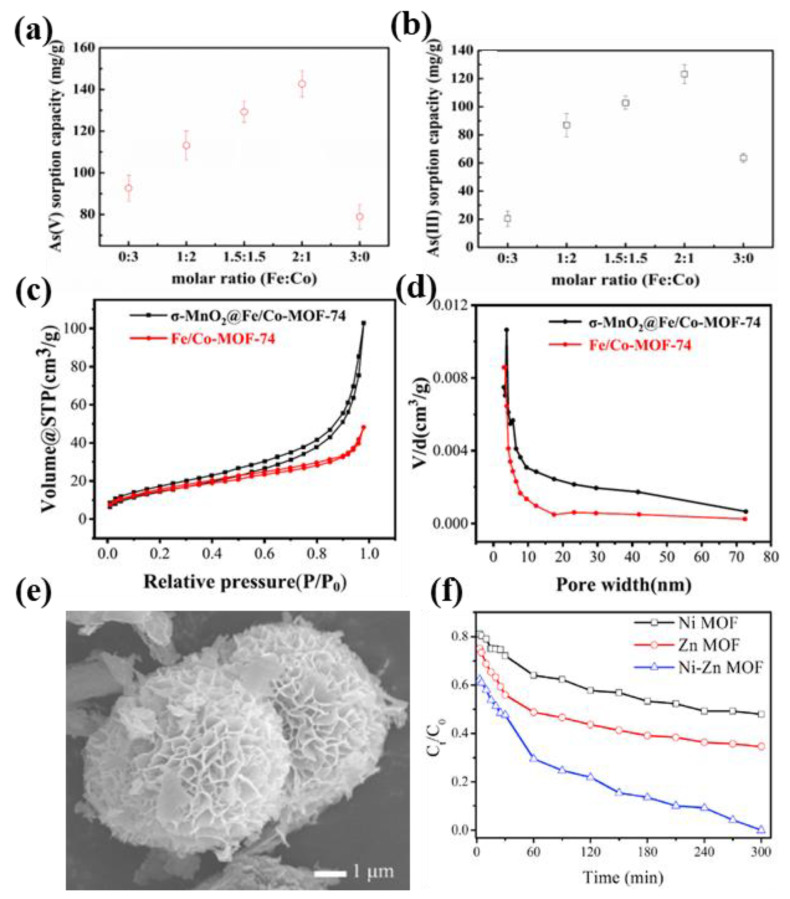
The effect of Fe/Co molar ratio on arsenic removal by Fe_2_Co_1_ MOF-74 adsorbent, (**a**) As^5+^ and (**b**) As^3+^ (Initial arsenic concentration = 100 mg/L, adsorbent dose = 0.5 g/L, temperature = 25 °C). Reproduced with permission from ref. [67]. Copyright 2019, Elsevier. (**c**) N_2_ adsorption–desorption isotherms and (**d**) pore width distribution of Fe/Co-MOF-74 and δ-MnO_2_@Fe/Co-MOF-74. Reproduced with permission from ref. [69]. Copyright 2021, Elsevier. (**e**) SEM image of NiZn MOF and (**f**) adsorption efficiency of Congo red on Ni MOF, Zn MOF, and NiZn MOF. Reproduced with permission from ref. [72]. Copyright 2019, Elsevier.

Sun and co-researchers constructed Fe-Co-based bimetallic MOF adsorbents with different Fe/Co molar ratios (Fe_x_Co_y_ MOF-74, where x:y = 0:3, 1:2, 1.5:1.5, 2:1, and 3:0) [67]. Microstructure analysis showed that the BET-specific surface area of bimetallic Fe_2_Co_1_ MOF-74 increased by about 20 times (147.82 m^2^/g) and the total pore volume increased by about 4 times (0.058 cm^3^/g) compared with that of monometallic Co MOF-74. At room temperature, compared to other Fe/Co molar ratio bimetallic MOF-based materials, the maximum adsorption capacity of Fe_2_Co_1_ MOF-74 for As^5+^ and As^3+^ increased significantly, reaching 292.29 mg/g (Figure 3a) and 266.52 mg/g (Figure 3b), respectively. The study displayed that the absorption capacity of Fe_2_Co_1_ MOF-74 to As^5+^ and As^3+^ was mainly attributed to the enhancement of specific surface area, total pore volume, pore structure, and nanomaterial size.

Zhang and colleagues designed an amorphous Fe/Mn bimetallic organic framework (FeMn-MOF-74), which effectively combined the low coordination active centers with the metal atoms at the high oxidation sites to achieve the overall efficient removal of As^3+^ [68]. When the molar ratio of Fe to Mn was 1.96, the synergistic effect between the active sites of Fe and Mn was the best, and the maximum adsorption capacity of the material for As^3+^ could reach 161.6 mg/g under laboratory conditions. The study confirmed that the synergistic effect between the components of bimetallic MOFs in amorphous form played a decisive role in promoting the adsorption of trivalent arsenic. Similarly, a fusiform FeCo bimetallic MOFs-based nanomaterial (δ-MnO_2_@Fe/Co-MOF-74) was successfully prepared by Yang et al., which exhibited high adsorption removal rates for As^3+^ in the pH range from 2 to 10 [69]. Compared with Fe/Co-MOF-74, the N_2_ adsorption–desorption curves (Figure 3d) and pore size distributions (Figure 3c) of MnO_2_@Fe/Co-MOF-74 were significantly ameliorated. At room temperature, the maximum adsorption capacity of δ-MnO_2_@Fe/Co-MOF-74 for As^3+^ was 300.5 mg/g, while that of Co-MOF-74 was 159.3 mg/g. The mechanism analysis explained that the strong coordination of Fe^3+^/Co^2+^ for As was the important reason for the significant improvement of adsorption performance for bimetallic MOFs-based nanocomposites, besides the functional groups of Fe/Co-O, Fe/Co-OH, and Mn-O.

#### 3.1.2. Organic Compound Adsorption

The continuous development of bimetallic MOF-based adsorbents with high physicochemical properties, high adsorption capacity and selectivity of organic matter, and low production costs is worth further exploration. Bimetallic MOFs are materials with a large surface area, high porosity, and multiple active sites that have a good development prospect in the adsorption of organic pollutants in water environments [70,71].

The introduction of a second metal into the monometallic MOF can improve the stability of the MOF as a whole and also enhance the interaction between the material and the targeted contaminant. Yang and Bai [72] prepared flower-like hierarchical NiZn MOF microspheres by hydrothermal method (Figure 3e) to improve the stability of the adsorbent and the adsorption capacity of organic pollutant Congo red. The result showed the adsorption capacity of NiZn MOF to Congo red increased to 460.90 mg/g (Figure 3f), while that of Zn MOF and Ni MOF was 276.60 mg/g and 132.20 mg/g, respectively. In addition, the density functional theory (DFT) calculation of NiZn MOF confirmed that the NiO, ZnO, and NiO-ZnO nanoclusters contained in the adsorbent were favorable for the adsorption of Congo red molecules. Similarly, Zheng et al. prepared a cobalt–zinc bimetallic MOFs-based nanomaterial (ZnCo-NPC) with a special three-dimensional porous structure [73], and the maximum adsorption capacity of the material was up to 320 mg/g for ornidazole (ODZ) in water. Adsorption mechanism analysis showed that ZnCo-NPC enhanced the capture capacity for ODZ molecules by intensifying electrostatic forces, hydrogen bonds, and π–π bonds.

Liu and co-team synthesized multi-component hollow and multi-shell Ni-Co bimetallic MOFs-based nanomaterials (Fe(OH)_3_@NiCo-LDH) to strengthen the interaction between unsaturated sites and Congo red molecules in the material’s skeleton structure [74]. Within 20 min, the maximum removal efficiency of Congo red by Fe(OH)_3_@NiCo-LDH reached 100%, and the saturated adsorption capacity reached 658.52 mg/g, significantly higher than that of Fe(OH)_3_@NiMg-LDH. The remarkable difference in adsorption capacity of Congo red by bimetallic MOFs-based materials with distinct structures (Fe(OH)_3_@NiCo-LDH is a double-shell structure, and Fe(OH)_3_@NiMg-LDH is a hollow tube structure) indicated that the binding strength between the adsorption sites of the material structure and goal molecules was different. The results demonstrated that the adsorption active sites formed by NiCo-MOFs-based materials were better than those formed by NiMg-MOFs-based materials.

However, another report also showed similar research content; the maximum adsorption capacity of the prepared bimetallic MOFs on Congo red was more than three times that of the above reports. The study focused on the adsorption properties of cobalt and iron bimetallic MOFs for CoFe-BDC-(1), which was synthesized in one pot, and CoFe-BDC-(2), which was prepared by the MOF-on-MOF method (that is, CoFe-MOFs were further structured on the basis of Fe-BDC) [75]. The maximum adsorption capacity of CoFe-BDC-(1) adsorbent for Congo red reached 1935.68 mg/g, while that of CoFe-BDC-(2), Fe-BDC, and Co-BDC was 1259.52, 775.19, and 628.93 mg/g, respectively. The SEM, TEM, and N_2_ adsorption–desorption analysis exhibited that CoFe-BDC-(1) had more defects than CoFe-BDC-(2), which could improve the adsorption and removal efficiency of anionic pollutants in water. The above two reports indicate that the design of an adsorbent for the same target pollutant should be fully considered from the aspects of synthesis process, metal type, material structure, and function.

**Table 1:** Recent advances of bimetallic MOFs-based nano-adsorbents for adsorption of pollutants in water.

Adsorbents	Pollutants	Concentration/mg/L	SSA ^a^ /m^2^/g	K ^b^ /min^−1^	k_max_^c^ /mg/g	Ref.
CuFe-BTC	U(Ⅳ)	50	-	-	354	[20]
NC@GC/CNTs	NaCl	1000	516.46	-	77.33	[53]
Fe doped HKUST-1	Pb^2+^	50	438.9	0.041	564.9	[58]
Co_x_Mg_1−x_-MOF-74	1-hexene	-	1135	-	152.7	[59]
NiFe-MOF	Crystal violet	400	9.2	0.003	395.9	[63]
	Tetracycline	400		0.003	568.1	
MIL-125(Ti)/MIL-53(Fe)/CNT@Alg	Tetracycline	50–300	273.77	-	294.12	[64]
NiCo-MOF@CMC	Tetracycline	30	-	-	624.87	[65]
Zinc/Iron mixed-metal MOF-74	Methylene blue	50	1280	-	370	[66]
	Methyl orange	50		-	239	
Fe-Co based MOF-74	As^5+^	1	147.82	-	292.29	[67]
	As^3+^	1		-	266.52	
Fe/Mn-MOF-74	As^3+^	5–50	45.82	-	161.6	[68]
δ-MnO_2_@Fe/Co-MOF-74	As^3+^	5–160	-	-	300.5	[69]
GO/Ni-MOF-199	Thiophene	500	777.88	0.494	50.38	[70]
ZnIn_2_S_4_-Nanosheet	Ciprofloxacin	100–1000	118.6	-	219	[71]
	Congo red	50		-	257	
NiZn MOF	Congo red	30	56.7	0.0089	460.90	[72]
CoFe-MOF	Congo red	0.072 mol/L	10.57	0.0088	1935.68	[75]
Cobalt/zinc-based MOF-74	Ornidazole	500	231	-	320	[73]
Fe(OH)_3_@NiCo-LDH	Prussian blue	100	187.90	0.055	658.52	[74]
Ag-Fe MOF	Cd^2+^	50–200	1150.3	0.539	265	[76]
	Cu^2+^			0.860	213	

^a^ SSA represents the specific surface area, ^b^ k is the rate of adsorption, ^c^ k_max_ is maximal adsorption capacity.

### 3.2. Catalysis

Catalysis is an important direction in the development of bimetallic MOF-based nanomaterials. In terms of the intrinsic catalytic mechanism of MOF-based nanomaterials as catalysts, inorganic nodes owned by MOFs themselves are the important basis for the catalytic properties of catalytic materials. In fact, during the catalytic process of inorganic nodes, the intensity of the electric field around metal ions would change, which could cause fluctuations in the coordination environment between metal ions and organic ligands, further inducing the collapse of the metal-organic framework. Therefore, compared with monometallic MOF-based catalysts, the design of bimetallic MOF-based catalysts is wise; that is, one highly active metal ion mainly acts as the catalytic center, and the other maintains the stability of the catalyst structure. At the same time, the construction of bimetallic MOF-based materials can effectively optimize the distribution of metal catalytic active sites, improving their stability, catalytic activity, and selectivity [76,77,78,79,80]. The recently catalyzed degradation of pollutants in the water environment by bimetallic MOF-based nanocomposites is summarized in Table 2.

#### 3.2.1. Catalytic Activation Degradation

The catalytic activation degradation process is based on the fact of activating H_2_O_2_, PMS, and PDS to produce highly active hydroxyl radicals (**•**OH) and sulfate radicals (SO_4_**^•^**^−^), which effectively act and degrade stubborn organic pollutants. Over the past decade, hundreds of studies have confirmed that the catalytic activity of SO_4_**^•^**^-^ is superior to that of **•**OH and that oxidants (PMS) with asymmetric structures are more conducive to activation than those (H_2_O_2_ and PDS) with symmetric structures. In response to the call for green, efficient, and sustainable development goals, heterogeneous catalysts have gradually replaced homogeneous catalysts, becoming the hot spot for catalytic activation and degradation of organic pollutants in water [81,82]. Among many heterogeneous catalysts, the bimetallic MOF-based nanocomposites, especially Co-based MOFs [83], that directly rely on the advantages of attainable and highly effective metal active sites and structures are regarded as efficient catalysts for catalytic degradation of pollutants in water.

Huang and co-workers prepared hierarchical CoFe LDH/MOFs nanorods with different cobalt and iron contents for urea oxidation in solution [84]. Compared with Co-MOF, the CoFe LDH/MOF catalyst had a larger surface activity, higher conductivity, and a better electron mass transfer path, making the material favorable to promote the catalytic reaction. The catalytic degradation of urea by bimetallic CoFe-MOFs-based materials confirmed that CoFe LDH/MOFs still had good recyclability after a long time of work, which was attributed to the improvement of the local electronic structure for the catalytic active site. Meanwhile, with the introduction of iron ions, the adsorption capacity, or controllability, of the catalyst for inorganic metal nodes and ligands gradually increased. Wang and colleagues synthesized layered Pd@ZnNi-MOF/GO catalysts to activate H_2_O_2_ degrading 3,7-bis(Dimethylamino)phenazathionium chloride [85]. The introduction of Pd atoms not only greatly improved the catalytic activity of the nanocomposite on H_2_O_2_, but also reinforced its own stability.

Yang and co-team synthesized porous magnetic bimetallic MOFs-based nanocrystal catalyst CoFe_2_O_4_ NC using a CoFe bi-MOFs template for degradation of bisphenol A (BPA) with activating PMS (Figure S3a) [86]. Compared with CoFe_2_O_4_ NC/BPA (only 4% BPA removal efficiency), Fe-MOF/PMS/BPA (5%), and Co-MOF/PMS/BPA (63%), the catalytic removal efficiency of BPA by the CoFe_2_O_4_ NC/PMS/BPA system was more than 97% in 60 min (Figure S3b), which illustrated that introducing iron ions into Co-MOF could significantly promote the activation of PMS and the catalytic degradation of BPA. The CoFe_2_O_3_ nanoparticle (CoFe_2_O_4_ NP) prepared by the traditional one-step hydrothermal method was also weaker than the CoFe_2_O_4_ NC in the degradation of BPA by activating PMS, and the BPA removal efficiency of the former was 69% under the same experimental conditions. These results indicate that the synergistic catalytic effects between Co^2+^ and Fe^2+^ are significantly different in terms of CoFe_2_O_4_ NP and CoFe_2_O_4_ NC with different structures.

Li and co-researchers synthesized a series of unique macaroon-like NbCo-MOF materials for heterogeneous catalyst activation of PMS [87]. The catalytic degradation performance of tetracycline (TC) in NbCo-MOF samples with Nb/Co ratios of 1:1, 1:2, 1:3, 1:4, 1:5, 2:1, and 4:1 was investigated. In the kinetic process of TC degradation by activating PMS, the catalyst samples with the Nb/Co ratio of 1:4 showed the highest catalytic activity (within 10 min, the removal efficiency of TC was close to 100%), while the catalytic activity of other samples ranged from 35% to 79% (Figure S3c). The anion effects of HCO_3_^−^, NO_3_^−^, C_2_O_4_^2−^, and Cl^−^ co-existing with TC were investigated in the NbCo-MOF/PMS system, and the results displayed that the presence of these anions hardly affected the catalytic degradation of TC (Figure S3d–g). In addition, NbCo-MOF exhibited excellent catalytic degradation of rhodamine B and tylosin tartrate. Within 30 min, the removal efficiency of these target molecules in water was greater than 98%. The research emphasized that the combined effect of the Nb^4+^/Nb^5+^ cycle and the Co^2+^/Co^3+^ cycle (Equations (1)–(6)) in the NbCo-MOF catalyst accelerated the regeneration of highly active sites and improved the electron transfer efficiency between the catalyst and PMS, thus effectively promoting the catalytic activity of the catalyst for degrading dissolved organic pollutants.

Nb^4+^ + HSO_5_^−^ → Nb^5+^ + SO_4_**^•^**^−^ + OH^−^ (1)

Nb^5+^ + HSO_5_^−^ → Nb^4+^ + SO_5_**^•^**^−^ + H^+^ (2)

Co^2+^ + HSO_5_^−^ → Co^3+^ + SO_4_**^•^**^−^ + OH^−^ (3)

Co^3+^ + HSO_5_^−^ → Co^2+^ + SO_5_**^•^**^−^ + H^+^ (4)

Nb^4+^ + Co^3+^ → Nb^5+^ + Co^2+^ (5)

Nb^5+^ + Co^2+^ → Nb^4+^ + Co^3+^ (6)

**Figure 4:**
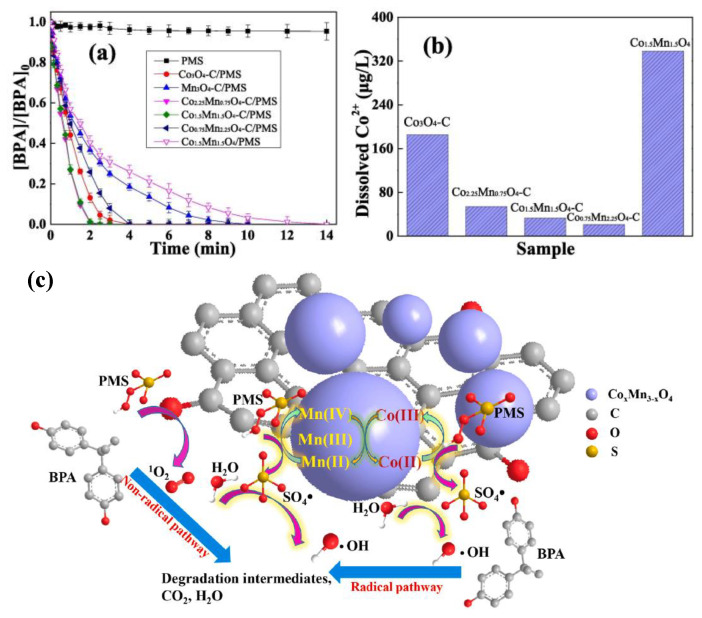
(**a**) BPA removal, (**b**) the corresponding Co^2+^ leaching by different catalysts. ([BPA]_0_ = 0.1 mM, [PMS]_0_ = 1.0 mM, [catalyst]_0_ = 0.1 g/L), and (**c**) mechanism illustration of BPA degradation in Co_1.5_Mn_1.5_O_4_-C/PMS system. Reproduced with permission from ref. [88]. Copyright 2022, Elsevier.

The multi-component reaction in bimetallic MOF-based materials that can be realized smoothly is based on the coordination of different active sites [88,89,90,91]. By adjusting the target metal ratio, Wang and co-members proved that the materials with spinel-structured CoMn-MOFs-based nanoparticles (Co_x_Mn_3−x_O_4_-C) possessed high catalytic activity and were constructed with a Co/Mn bimetallic organic framework as a template [88]. A series of Co_x_Mn_3−x_O_4_-C samples showed substantial performance in activating PMS to degrade bisphenol A (BPA), and the Co_1.5_Mn_1.5_O_4_-C had the best performance. The material could completely degrade 0.1 mM BPA in a very short time (3 min) (Figure 4a). The stability experiments measured the leaching amount of cobalt ions for prepared catalysts during the catalytic process (Figure 4b), which strongly confirmed that the structure of Co_1.5_Mn_1.5_O_4_-C had high fixability on metal ions during working. Common inorganic anions (such as HCO_3_^−^, H_2_PO_4_^−^, NO_3_^−^, and Cl^−^) also had little effect on the catalytic degradation of BPA by the Co_1.5_Mn_1.5_O_4_-C/PMS system, but humic acid had a significantly negative effect. Finally, the mechanism analysis highlighted that there are two pathways for the catalytic degradation of BPA (Figure 4c). One is that the active sites of cobalt and manganese directly catalyze PMS to form **•**OH and SO_4_**^•^**^−^ radicals working on BPA molecules, while the other is that the carbonyl group (C=O) under the action of electron transport for the carbon framework in the catalyst interacts with PMS to produce singlet oxygen ^1^O_2_ attacking BPA.

#### 3.2.2. Photocatalytic Degradation

In recent years, bimetallic MOFs-based materials have gradually developed various excellent environmental photocatalysts [92,93,94,95,96], which could be used as prominent photocatalytic reaction active agents for the degradation of organic pollutants in water. It is generally acknowledged that titanium-based and zinc-based metal organic frameworks have become hot spots in the field of photocatalysis due to their unique absorption and conversion properties of light [97,98,99]. Another metal combined with Ti or Zn to form the bimetallic MOFs could significantly promote the photocatalytic activity of the materials.

Li et al. investigated the application of TiIn-MOF catalysts with different proportions for adsorption and photocatalytic degradation of BPA [100]. Among them, the heterogeneous metal clusters formed by TiIn-MOF(1:1) could well regulate the surrounding electronic structure and significantly improve the absorption intensity of visible light (Figure S4a). Moreover, the lowest optical bandgaps (2.60 eV) were found among the synthesized bimetallic MOF-based catalysts (Figure S4b). After 30 min of dark treatment, the adsorption removal efficiency of BPA by TiIn-MOF(1:1) reached 38%, and the adsorption performance was about twice that of Ti-MOF. Subsequently, under 300 W xenon lamp irradiation, the degrading efficiency of BPA was 100% by TiIn-MOF(1:1) after 20 min, and the degradation rate was twice that of Ti-MOF. In addition, the synergistic effect of TiIn-MOF(1:1) could promote the production of **•**O_2_^−^, hole (h^+^) and ^1^O_2_ in the photocatalytic process by a large margin. However, a part of the pores and active sites for the directly synthesized Ti-based MOFs were masked by precursor molecules (organic ligands, organic solvents, and the like), which resulted in a decrease in the activity of the catalyst. Therefore, Wang et al. performed a thermal treatment of the synthesized ZnTi-MOF (Figure S4c) to prepare a C-doped MOFs-based bimetallic photocatalyst that displayed highly efficient visible light absorption [101]. After 45 min irradiation by a 200 W xenon lamp, the catalytic degradation efficiencies of all C-dope ZnTi-MOF samples were better than those of untreated samples.

Amine-functionalized bimetallic MOFs have been successfully applied in the preparation of photocatalytic materials. Amination can not only expand the visible light absorption of MOFs-based materials but also well protect the oxygen-containing functional groups on the surface, which enhances the utilization rate of light by the photocatalyst, adding to its own oxidation resistance [102,103]. Wang et al. used FeTi-MOF-NH_2_ as a photo-Fenton catalyst to degrade chrysoidine at 300 W xenon lamp irradiation [103]. Within 10 min of the reaction starting, the photo-catalysis ability of Fe/Ti-MOF-NH_2_(3:1) degrading the goal pollutant increased to 100%, while FeTi-MOF-NH_2_(1:1) and FeTi-MOF-NH_2_(1:3) were only about 40%. The heterojunction structure of FeTi-MOF-NH_2_(3:1) could effectively slow down the photocatalytic electron-hole pair recombination rate and improve the effective photocatalytic e^-^-h^+^ pairs so as to exhibit highly efficient catalytic performance (Equations (7)–(11)).

Fe/Ti-MOF-NH_2_(3:1) + *hv* → Fe/Ti-MOF-NH_2_(3:1) (e^−^ + h^+^) (7)

S_2_O_8_^2−^ + e^−^ → SO_4_**^•^**^−^ + SO_4_^2−^ (8)

O_2_ + e^−^ → O_2_**^•^**^−^ (9)

2O_2_**^•^**^−^ + 2H_2_O → 2**•**OH + 2OH^−^ + O_2_ (10)

Chrysoidine + SO_4_**^•^**^−^, **•**OH, O_2_**^•^**^−^, h^+^ → degradation products (11)

Zhong and co-workers designed an AB-type heterojunction photocatalyst, ZnO/Ni_0.9_Zn_0.1_O using MOF-NiZn as the intermediate [104]. It is worth noting that during the pyrolysis process of MOF-NiZn, some Ni metal nodes were replaced by Zn to form Ni_0.9_Zn_0.1_O. Compared with NiO (3.11 eV), the hybrid state of Ni_0.9_Zn_0.1_O (2.98 eV) had a lower band gap. Therefore, ZnO/Ni_0.9_Zn_0.1_O only needed lower photon energy to excite and generate electrons and holes in visible light, displaying better photocatalytic performance. The photocatalytic degradation efficiency of ZnO/Ni_0.9_Zn_0.1_O for phenothiazine could reach more than 97% in 60 min.

**Figure 5:**
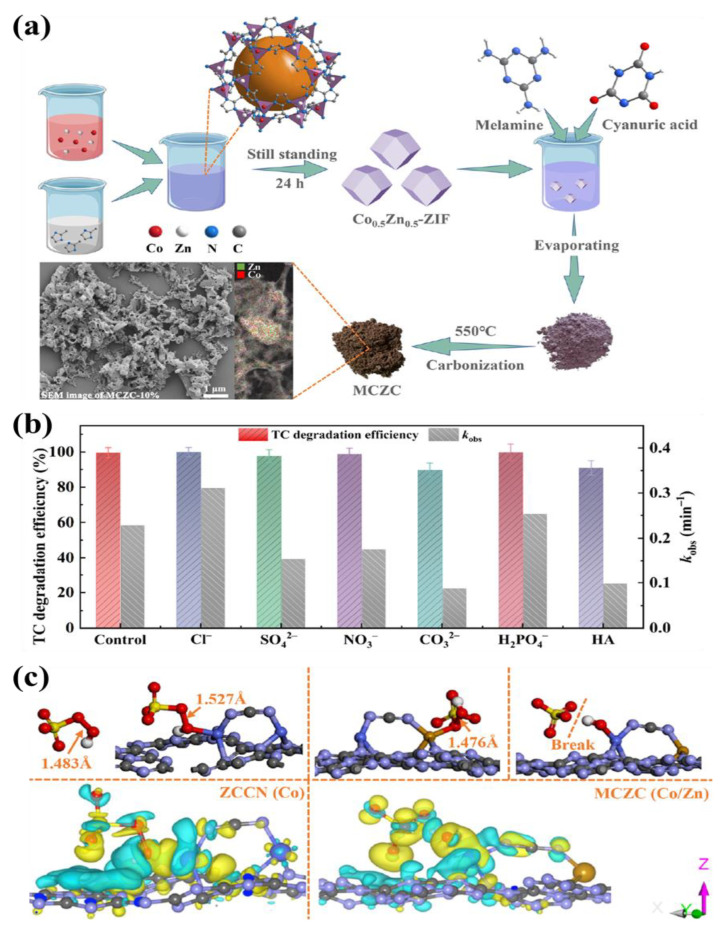
(**a**) Synthesis of MCZC catalyst; (**b**) Influences of inorganic ions and HA on TC degradation over MCZC-10% with the concentrations of inorganic ions and HA being 20 mM and 10 mg/L, respectively; (**c**) Geometric optimizations of PMS adsorbed onto the Co site of ZCCN and MCZC catalysts corresponding electron density differences (Yellow and lake-blue regions represent electron-rich and electron-defective areas, respectively). Reproduced with permission from ref. [105]. Copyright 2023, Elsevier.

Li et al. constructed CoZn-ZIF as a porous, non-bonding nano-catalyst CoZn-N-C (MCZC) using thermal etching technology (Figure 5a), which could enhance the electron transfer between the Co active site and the Zn active site and construct the unsaturated metal sites of Co and Zn atom pairs to the maximum extent [105], improving the synergistic effect between different active sites of the catalyst. When the photocatalytic time is 24 min, the maximum removal efficiency of TC in the MCZC/PMS/Visible-light system was 99.6% (the mineralization rate was 55.8%), while that in ZCCN (ZCCN was the thermal etching product of ZIF-67) was only 74.9% without visible-light irradiation. A variety of anions (including Cl^−^, SO_4_^2−^, NO_3_^−^, CO_3_^2−^ and H_2_PO_4_^−^) and natural organic matter (HA) did not significantly affect the maximum degradation efficiency of the MCZC/PMS/Vis/TC system, but the removal rates were significantly different. The presence of Cl^-^ and H_2_PO_4_^−^ increased the catalytic rates of MCZC, while the rest showed inhibition (Figure 5b). The internal mechanism of the catalytic performance improvement for MCZC was explained mathematically by density functional theory (Figure 5c). When PMS was adsorbed on the Co active site at the photocatalyst surface of ZCCN or MCZC, the O-O bond length of PMS would be outstandingly stretched (from 1.483 Å or 1.476 Å to 1.527 Å) at visible-light irradiation driving. Thus, as for MCZC, the PMS was more easily activated by photogenerated electrons to produce SO_4_**^•^**^−^ and **•**OH.

**Table 2:** Recent forefront of bimetallic MOFs-based nano-catalysts for catalytic degradation of pollutants in water.

MOFs Precursors	Catalysts	Pollutants	Concentration	PMS (Dosage)	Light Source	Time /min	R ^d^	Ref.
ZIF-67, MOF-74(Ni)	Co-MOF/Ni-nanoparticles/g-C_3_N_4_	Venlafaxine	10 mg/L	0.4 mM	-	120	91.38	[83]
ZIF-67, MIL-100(Fe)	Co-MOF/Fe-nanoparticles/g-C_3_N_4_						100	
ZnNi-MOF	Pd@ZnNi-MOF/GO	3,7-bis(Dimethylamino)phenazathionium chloride	10 mg/L	-	-	8	95	[85]
Titanium isopropoxyde, In(NO_3_)_3_·9H_2_O	In/Ti-MOF	Bisphenol A	50 mg/L	-	300 W Xenon lamp	20	100	[100]
CoMn-MOF-74	Co_x_Mn_3-x_O_4_	Bisphenol A	0.1 mM	1.0 mM	-	14	100	[88]
ZnFe-MOF	ZnFe_2_O_4_/CNTs	Bisphenol A	50 mg/L	0.2 g/L	-	15	100	[33]
CoFe-MOF	CoFe_2_O_4_	Bisphenol A	45 µM	0.45 mM	-	60	97	[86]
Co(NO_3_)_2_·6H_2_O, Cu(NO_3_)_2_·3H_2_O 2,5-dihydroxy-1,4-benzene dicarboxylate	CuCo-MOF-74	Phenothiazine	0.2 mM	2.0 mM	-	30	100	[89]
MOF-NiZn	ZnO/Ni_0.9_Zn_0.1_O-82	Phenothiazine	20 mg/L	-	UV-light	60	97.4	[104]
Co(NO_3_)_2_·6H_2_O, C_10_H_5_NbO_20_, 2-aminoterephthalic acid	NbCo-MOF	Phenothiazine	20 mg/L	300 mg/L	-	30	100	[87]
		Tylosin tartrate					98.4	
		Tetracycline					99.7	
		Rhodamine B					99.7	
ZIF-8, ZIF-67	Co/ZIF-8	Rhodamine B	50 mg/L	150 mg/L	-	90	85	[77]
Co, Zn-MOF	Co@C-NCNTs	Norfloxacin	30 mg/L	0.5 mM	-	30	92	[90]
Co(NO_3_)_2_·6H_2_O, Ni-MOF, 1,3,5-tricarboxylic acid	NiCo-LDH	Red reactive	0.1 mM	3 mM	-	10	89	[91]
NiCe-MOF	NiO/CeO_2_	Sodium p-dimethylamino-azobenzene sulfonate	10 mg/L	-	125 W Mercury lamp	60	97	[92]
BiFe-MOF	BiFeO_3_	Naproxen	10.0 µM	0.10 mM	400 W Xenon lamp	40	95.5	[95]
CoCl_2_·6H_2_O, FeCl_3_·6H_2_O, CeO_2_, terephthalic acid	MIL-53(Fe/Co)/CeO_2_	Atrazine	10 mg/L	250 mg/L	80 W lamp	60	99	[96]
MIL-88B/Zn	ZnFe_2_O_4_/Fe_2_O_3_	Ciprofloxacin	10 mg/L	-	300 W Xenon lamp	180	96.5	[99]
ZnTi-MOF	C-doped ZnO/TiO_2_	Basic Rhodamine	10 mg/L	-	200 W Xenon lamp	45	94	[101]
FeTi-MOF	FeTi-MOF-NH_2_	Chrysoidine	50 mg/L	14 mM	300 W Xenon lamp	10	100	[103]
CoCl_2_·6H_2_O, FeCl_3_·9H_2_O, 2-aminoterephthalic acid	FeCo/N MOF	Tetracycline	50 mg/L	5 mM	-	150	99	[79]
ZIF-CoZn	Co-Zn-N-C (MCZC)	Tetracycline	10 mg/L	1 mM	-	24	99.6	[105]
		Ofloxacin					98.4	
		Norfloxacin					97.6	
		Phenol					97.7	

^d^ R is maximum degradation efficiency.

### 3.3. Detection

Bimetallic MOF-based materials possess high electron mobility, structural adjustability, and chemical stability, which have been widely used in the detection of target ions or molecules, especially as sensors [106,107,108,109,110]. Compared with the monometallic MOF-based electrode, the bimetallic MOF-based electrode sensor has the advantages of stronger high-temperature resistance, higher sensitivity, better selectivity, and a shorter response time [111,112,113]. The bimetallic MOFs are composed of two different metal cations or metal cations’ aggregates bonded with organic ligands to form functional materials with high specific surface area, porosity, and structural morphology stability, which is a new sustainable-development form based on single metal MOF materials. At present, a variety of sensors with special selective performance have been developed based on bimetallic MOFs-based materials and are popularly applied in the detection of targeted ions and molecules, including glucose [114,115], bisphenol A [116], Fe^3+^ [117], miRNA [118], antibiotics [119], acetone [120], organophosphate [121], and so on. The bimetallic MOF-based nanomaterials’ latest progress in detecting pollutants in environmental water is summarized in Table 3.

**Figure 6:**
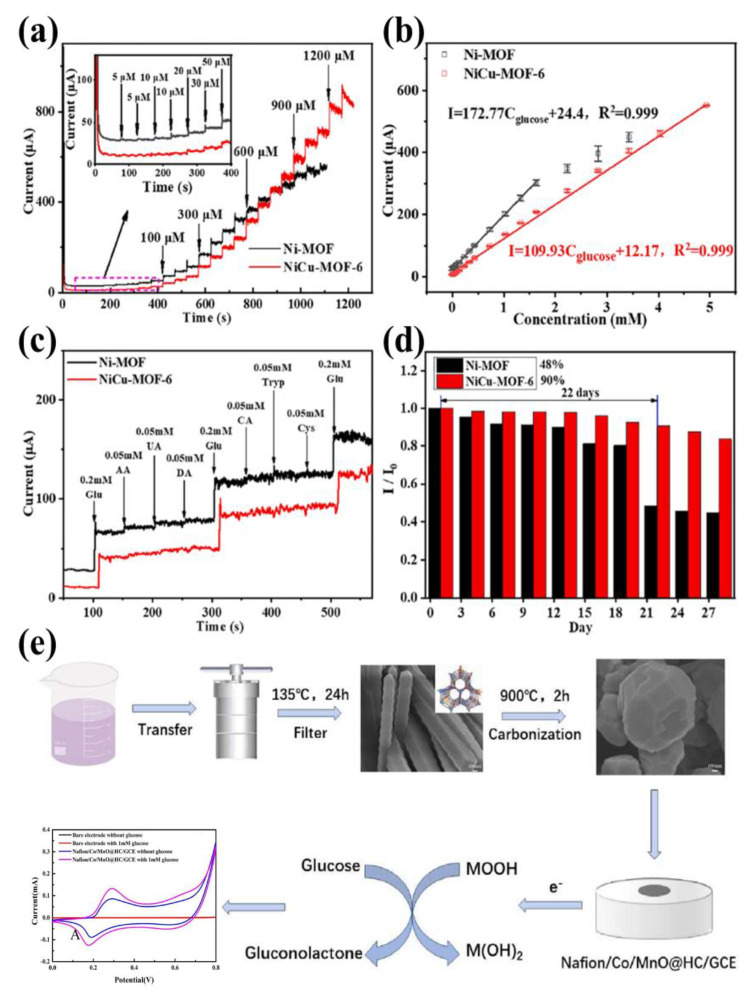
(**a**) Amperometric responses of Ni-MOF/GCE and NiCu-MOF-6/GCE upon successive addition of glucose (Inset shows the magnified amperometric response to glucose at lower concentrations); (**b**) The corresponding calibration curves of Ni-MOF/GCE and NiCu-MOF-6/GCE; (**c**) Anti-interfering capabilities of Ni-MOF/GCE and NiCu-MOF-6/GCE in the presence of glucose and other possible interference; (**d**) Stability of Ni-MOF/GCE and NiCu-MOF-6/GCE in 0.1 M NaOH solution containing 0.6 mM glucose. Reproduced with permission from ref. [114]. Copyright 2021, Elsevier. (**e**) Scheme showing the synthesis of Co/MnO@HC and modification of working glassy carbon electrode. Reproduced with permission from ref. [115]. Copyright 2022, Elsevier.

Pan and co-workers synthesized NiCu-MOF/GCE based on a glassy carbon electrode supported by 2D/3D hierarchical NiCu-MOF, which showed great electrocatalytic performance for a non-enzyme glucose sensor [114]. NiCu-MOF/GCE electrode sensors exhibited a favorable linear response to glucose in the range of 20 µM~4.93 mM, which was three times that of Ni-MOF/GCE (5 µM~1.63 mM) (Figure 6a,b). The NiCu-MOF/GCE’s performance advantage was not only manifested in the width of response to glucose but was also outstanding in anti-interference ability and stability. When the distractions of uric acid, citric acid, ascorbic acid, and Cl^−^ coexisted, the responsiveness of Ni-MOF/GCE and NiCu-MOF/GCE for glucose was deeply explored (Figure 6c). The results showed that the detection performance of NiCu-MOF/GCE for glucose was almost unaffected in the presence of high concentrations of Cl^−^, while Ni-MOF/GCE decreased by 22%. In addition, Figure 6d also reflected that after continuous operation for up to 22 days, the electrode performance of NiCu-MOF/GCE only went down 10%, while that of Ni-MOF/GCE under the same conditions reduced almost 52%. Another more efficient enzyme-free glucose bimetallic MOFs-based sensor (Co/MnO@HC) was successfully prepared (Figure 6e) [115]. Chronoamperometry and cyclic voltammetry evaluated that the Co/MnO@HC sensor had high electrochemical catalytic performance in low concentration glucose (50~900 µM) and high concentration glucose (1.9~6.9 mM) solutions, and the detection limit of the electrode material was 1.31 µM.

Huang and co-researchers prepared six self-assembled multilayer M-N-MOFs-based (M=Ce, Ni, Zn; N=Co, Ni) carbon nanotubes loaded on glassy carbon electrodes (M-N-MOF/MWCNTs/GCE) and applied them to the detection of BPA in drinking water [122]. Among the above six synthetic bimetallic MOFs, CeZn-MOF-based electrodes displayed the best catalytic performance and the highest oxidation peak current value (more than 40 µA) for BPA in aqueous solution. Nyquist plots further reflected the electrode impedances of CeZn-MOF/MWCNTs/GCE, CeZn-MOF/GCE, MWCNTs/GCE, and GCE, which were 34.64, 138.2, 84.26, and 103.9 Ω, respectively. The results demonstrated that CeZn-MOF/MWCNTs/GCE electrodes had the highest charge transfer efficiency in operation. Taking advantage of electrochemical impedance spectroscopy (EIS) and differential pulse voltammetry (DPV), the working range of CeZn-MOF/MWCNTs/GCE electrodes for BPA was determined from 0.1 to 100 µmol/L along with a detection limit of 7.2 nmol/L.

Wu and Huang synthesized an MgZn MOFs material with fluorescent characteristics: MgZn(1, 4-NDC)_2_(DMF)_2_ (1, 4-NDCH_2_ = 1, 4-naphthalene dicarboxylic acid), which was constructed by the chain of [-Mg-(COO)_2_-Zn-(COO)_2_-]_n_, exhibiting the two-color conversion fluorescence from green to blue [117]. The phenomenon was due to the change in interaction force and relative position between metal ions and organic ligands during the synthesis of bimetallic MOF-based materials, which led to the unique fluorescence behavior of the materials. In fact, MgZn(1, 4-NDC)_2_(DMF)_2_ could exhibit different fluorescence intensities because the fluorescence energy released by MgZn(1, 4-NDC)_2_(DMF)_2_ would be absorbed by metal ions or molecules. Therefore, MgZn(1, 4-NDC)_2_(DMF)_2_ showed excellent detection performance for Fe^3+^ (0–100 µM, Cu^2+^ (0–150 µM, CrO_4_^2−^ (0–600 µM) and CS_2_.

**Figure 7:**
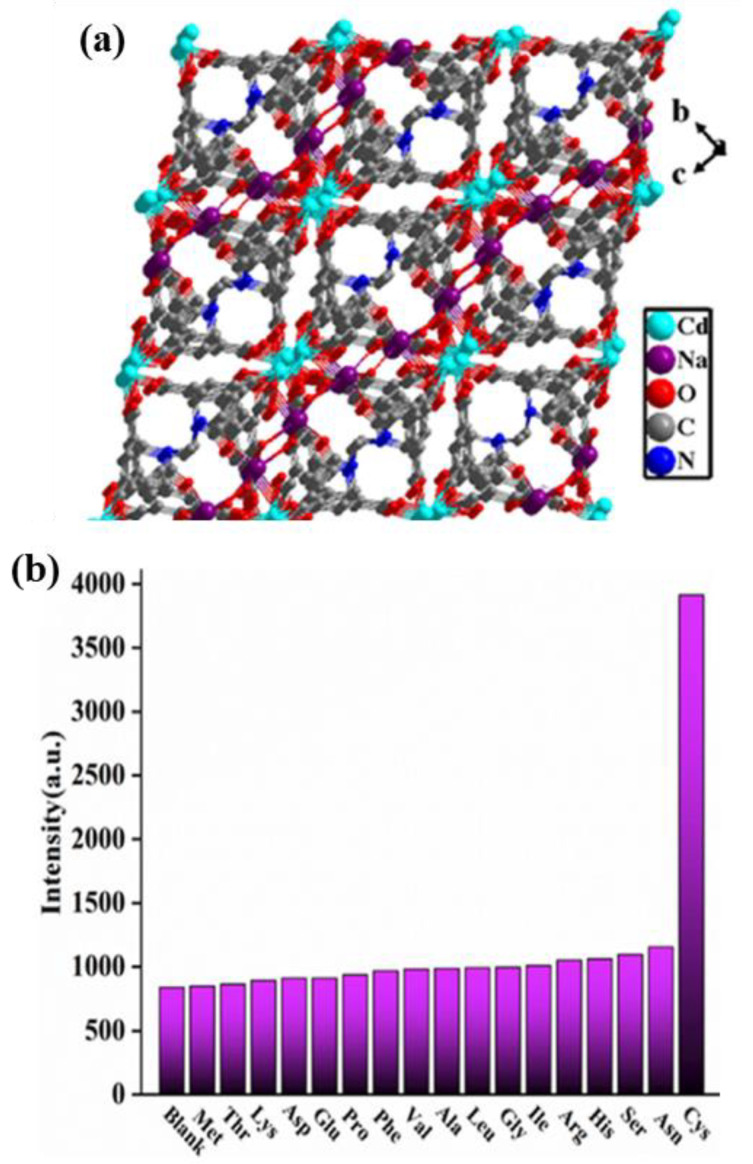
(**a**) The 3D framework of Cd_6_Na_4_(L)_4_(DMF)_2_(µ-H_2_O)_3_; and (**b**) The emission intensities of Cd_6_Na_4_(L)_4_(DMF)_2_(µ-H_2_O)_3_ in different amino acids. Reproduced with permission from ref. [119]. Copyright 2022, Elsevier.

Wang and co-workers have designed another high-efficiency fluorescent bimetallic MOFs-based sensor, Cd_6_Na_4_(L)_4_(DMF)_2_(µ-H_2_O)_3_ (H_4_L = 1,1′-ethylbiphenyl-3,3′,5,5′-tetracarboxylic acid) (Figure 7a), for the detection of ODZ, cysteine (Cys), TC, and metronidazole (MDZ) in water [119]. Interestingly, Cd_6_Na_4_(L)_4_(DMF)_2_(µ-H_2_O)_3_ exhibited different detection characteristics when working. On the one hand, a fluorescence quenching effect was displayed on the detection of ODZ (the detection limit was 0.32 µM), TC (0.11 µM), and MDZ (0.14 µM). On the other hand, a fluorescence-enhancing effect was revealed during the process of recognizing Cys (19.7 nM). When ODZ, TC, or MDZ were presented in water, the ligands of Cd_6_Na_4_(L)_4_(DMF)_2_(µ-H_2_O)_3_ preferentially transferred energy to target molecules ODZ, TC, or MDZ, while the energy transferring to other receptors was inhibited, thereby reducing fluorescence intensity, which manifested as selective recognition of ODZ, TC, or MDZ. The fluorescence enhancement effect was the result of a synergistic effect between Cys and the porous structure of CdNa-MOFs. Targeting experiments (Figure 7b) and interference tests of 17 amino acids (Met, Thr, Lys, and the like) proved that Cd_6_Na_4_(L)_4_(DMF)_2_(µ-H_2_O)_3_ sensor owed characteristic detection performance to Cys, and cyclic tests further highlighted that the sensor possessed the properties of maintaining good stability and recycling.

**Table 3:** Recent progress of high-sensitivity bimetallic MOF-based sensors for detecting pollutants in water.

MOFs Precursors	Sensors	Goals	Linear Range /µM	LOD ^e^/nM	Response Time/s	Ref.
AgZn-MOF	AgZn-MOF	HCV-RNA	1 fM~100 nM	0.64 fM	3.5 h	[37]
ZnFe-MOF	Fe_2_O_3_@ZnFe_2_O_4_	Acetone	1.4~300	-	7.6	[35]
Zn-MOF-5, Fe(NO_3_)_3_·9H_2_O	ZnFe_2_O_4_/Fe-ZnO	Acetone	30.8–200 mg/L	-	4.7	[111]
CuCo-MOF	CuO_x_@Co_3_O_4_ core-shell nanowires	Glucose	0.1~1300.0	36	1	[107]
CuNi-MOF	M/MO-800@C	Glucose	0.1~2200	60	5	[108]
Ni(NO_3_)_2_·6H_2_O/Cu (NO_3_)_2_·3H_2_O	NiCu-MOF/GCE	Glucose	20~4930	15,000	-	[114]
MnCo-MOF-74	Co/MnO@HC/GCE	Glucose	50~900, 1900~6900	1310	-	[115]
NiCo-MOF	NiCo-MOF/Ag/rGO/PU	Glucose	10~660	3280	12	[123]
FeCl_3_·6H_2_O, EuCl_3_·6H_2_O, 2-aminoterephthalic acid	FeEu-MOF	Alkaline phosphatase	0~250	0.6	-	[110]
CeZn-MOF	CeZn-MOF/MWCNT/GCE	Bisphenol A	0.1~100	7.2	125	[122]
Ce(NO_3_)_2_·6H_2_O, Ni(NO_3_)_2_·6H_2_O, 1,3,5-benzenetricarboxylic acid	Ce-Ni-MOF/GCE	Bisphenol A	0.1~100	7.8	150	[116]
EuCl_3_, TbCl_3_, CTP-COOH	Versatile Eu^3+^/Tb^3+^-MOFs	Fe^3+^	20~250	3860	<10	[124]
EuCl_3_·6H_2_O, TbCl_3_·6H_2_O, protonated pyridine-3,5-dicarboxylic acid sulfate ligand	Eu_x_Tb_1-x_-MOFs	Fe^3+^	30~120	10,000	-	[125]
Mg(NO_3_)_2_, Zinc acetate, 1,4-naphthalene dicarboxylic acid	MgZn(1,4-NDC)_2_(DMF)_2_	Fe^3+^	0~100	-	-	[117]
		Cu^2+^	0~150	-	-	
		CrO_4_^2−^	0~600	-	-	
CdNa-MOF	Cd_6_Na_4_(L)_4_(DMF)_2_(μ-H_2_O)_3_	Ornidazole	0~65	320	20	[119]
		Cysteine	0~2	19.7	20	
		Tetracycline	0~30	110	20	
		Metronidazole	0~150	140	20	
		Glucose	5~35	3500	30	
K_3_[Fe(CN)_6_], MnCl_2_·4H_2_O, terephthalic acid	MnFe-MOF	Organophosphate	0.001~0.1	0.85	120	[121]
ZnNi-MOFs	ZnNi MOF microspheres	Adenosine	0.0001~100 ng/mL	20.32 fg/mL	-	[126]
BMZIF67@MWCNTs	Co-N_x_-C@MWCNTs	Pb^2+^	2~60 µg/L	0.7 µg/L	120	[127]
Fe-MOF, TbCl_3_·6H_2_O	TbFe-MOFs	Carbohydrate antigen	0.01~200	58	-	[128]
ZIF-67@ZIF-8-P	CoP@C/NCS/GCE	Dopamine	5~400	30	200	[129]
NiCo-MOF	NiCo-MOF@C	*Helicobacter pylori*	10~10^7^ CFU./mL	1 CFU./mL	1200	[130]
FeCu-MOF	MOF(Fe-Cu)-ChOx	Choline	20~200	6	1200	[131]
CsCe-MOF	CsCe-MOF/GCE	Tryptophan	0.25~331	140	-	[132]
NiIn-MOF	Au-NiO/In_2_O_3_ hollow microspheres	Toluene	5~100 µg/L	5.1 µg/L	1	[133]
UiO-66, AgNO_3_, PdCl_2_, terephthalic acid, 2- aminoterephthalic acid	AgPd@UiO-66-NH_2_	4-Nitrophenol	100~370	32	60	[134]

^e^ LOD is limit of detection.

## 4. Summary and Outlook

### 4.1. Summary

The adjustable advantages of MOFs in morphology, structure, and chemical properties have made them a potential force for adsorption and catalytic treatment of polluted water and the detection of trace pollutants. Bimetallic MOFs increase the complexity of MOF-based materials on the basis of monometallic MOFs, including morphology, structure, catalytic properties, electron conduction, and stability. Direct synthesis and post-treatment have been widely adopted to prepare bimetallic MOF-based nanocomposites in order to maximize the efficiency of adsorption removal, catalytic degradation, and recycling.

The formation of heterojunctions and Schottky junctions significantly and effectively promoted electron transfer between the two metal atoms, thus enhancing the charge recombination cycle and improving the stability of the materials. In order to better explore the structure, composition, and arrangement of heterometals in bimetallic MOF-based materials, a variety of characterization techniques need to be fully utilized and combined. At present, the existing research lacks detailed and effective means to determine the arrangement of metals in the framework. However, the effective identification of metal ion arrangements in bimetallic MOFs-based materials is critical to understanding performance improvement in heterogeneous metal mixing modes.

Bimetallic MOF-based nanomaterials with complex structure and composition usually exhibit better performance than monometallic MOFs. MOFs composed of different metal centers can form bimetallic MOFs with different morphologies, structures, and functions through different preparation processes. Hydrothermal, solvothermal, and one-pot synthesis have become the basic strategies for bimetallic MOF synthesis, while ball-milling, pyrolysis, and other post-treatment methods have gradually developed into mainstream methods for the synthesis of new MOF-based materials. The synthesis process of new MOFs-based materials is usually accompanied by the formation of heterometal substitution and defective crystal structure so as to adjust the electronic structure, pore structure, and stability of bimetallic MOFs-based materials, leading to new physical adsorption and chemical catalytic properties.

At present, the effective synthesis and design of bimetallic MOF-based nanocomposites with various physical structures and chemical properties have been favored by many applications, including adsorption, catalysis, and the detection of pollutants in water environments. However, the sustainable development of bimetallic MOF-based materials is still limited by the following aspects: (a) low stability, (b) easy plugging of active sites and pore structures, (c) poor electrical conductivity, (d) unclear interaction mechanisms between bimetals, and (e) inconvenient recovery.

### 4.2. Outlook

A large number of studies on the characterization, recycling, and recovery of bimetallic MOF-based composites have been performed in detail and confirmed that the bimetallic MOF-based composites are more stable than the original MOFs.

Bimetallic MOF-based nanomaterials have more available active sites, relatively good stability, and electrical conductivity, which makes their applications in the fields of adsorption, catalysis, and detection under complex environmental conditions have stronger core competitiveness.

Although great progress has been made in the design of bimetallic MOFs as multifunctional materials, there are still some problems, such as the uneven distribution of metal particles, the absence of available active sites, and the difficulty of accurately controlling pore structure. In order to solve these problems as much as possible, studies using MOFs as precursors or templates are gradually developing. For example, the ball-milling method is used to control the mixing of the two precursor MOFs more fully so as to reduce the aggregation of bimetals in MOFs during the pyrolysis process. On the other hand, the doping of nonmetallic compounds can also enhance the toughness of bimetallic MOF-based nanomaterials. These strategies have improved the physical and chemical properties of bimetallic MOFs to a certain extent, but they still deserve further exploration.

The leaching of metal ions is an urgent problem to be solved in the application of bimetallic MOF-based materials in water treatment. The highly electronegative elements contained in the organic ligands can not only effectively fix metal ions in the synthesis of bimetallic MOFs-based materials, reduce ion leakage in the working process of nanomaterials, but also further optimize the pore structure and electron transport properties of the materials.

On the one hand, for the reaction mechanism and degradation path of pollutants in heterogeneous catalytic oxidation processes, many researchers only evaluated the mineralization degree of organic pollutants from the perspective of total organic carbon without considering the deeper carbon balance. In future studies, more effective evaluation methods can be considered to explain the conversion of pollutants between organic carbon and inorganic carbon. On the other hand, although bimetallic MOF-based nanocomposites have outstanding performance in removing pollutants from water environments, the comprehensive safety and ecological risk assessment of the materials still needs to be further studied.

Despite the fact that bimetallic MOF-based nanocomposites still face many challenges in water treatment, their rapid development in adsorbents, catalysts, and sensors (electrodes) in recent years indicates that they have huge potential for growth. In future research, it is expected to develop bimetallic MOF-based materials that are more efficient, cheaper, and environmentally friendly so that functional materials for water treatment can be realized with both structure and performance.

## Supplementary Materials

The following supporting information can be downloaded at: https://www.mdpi.com/article/10.3390/nano13152194/s1, Figure S1: (a) Schematic of the synthesis mechanism of bimetallic CoNi-MOFs; (b) Schematic drawing of the NiZn-MOFs nanostructure. Figure S2: (a) Diagrammatic synthesis of CoMo-MI-T; (b) CoNi@C is synthesized via a one-pot solvothermal and pyrolysis reaction; (c) Preparation procedure of CoCe-MOF. Figure S3: The schematic diagram of the preparation process for CoFe2O4 NC and the catalytic degradation of BPA by activating PMS and (b) removal efficiency of BPA in different reaction systems within 60 min. Effect of (c) Nb/Co ratio (catalyst = 0.01 g; TC = 100 mL, 0.02 g/L; PMS = 0.3 g/L; pH = 6.02; temperature = 25 °C; the dotted line represents the instability of Co-MOF), (d) Cl−, (e) NO3−, (f) HCO3− and (g) C2O42- on TC removal by NbCo-MOF (Nb:Co = 1:4). Figure S4: (a) UV–vis DRS spectra and (b) the band gap energy of as-prepared samples. (c) The process of preparation for ZnTi-MOF and photocatalytic degradation of basic Rhodamine.

## Nomenclature

BET	Brunauer-Emmet-Teller
BMOF	Bimetallic Metal Organic Framework
BPA	Bisphenol A
CC	Carbon Cloth
CoMo-MI	Cobalt-Molybdenum Bimetallic MOFs
Co-Mo-MI-T	Cobalt-Molybdenum Bimetallic MOF-based Carbon and Nitrogen Nanomaterials
CV	Cyclic Voltammetry
CNTs	Carbon Nano Tubes
Cys	Cysteine
DFT	Density Functional Theory
DMF	Dimethyl Formamide
DRS	Diffuse Reflectance Spectroscopy
DPV	Differential Pulse Voltammetry
EIS	Electrochemical Impedance Spectroscopy
Fe@NC-800/AG	Fe-Doped Nitrogen Carbon/Aerogel
GCD	Galvanostatic Charge/Discharge
GO	Graphene Oxide
LDH	Layer Double Hydroxide
MCZC	Cobalt/Zinc coordinated hollow carbon nitride
MDZ	Metronidazole
MIL	Materials of Institute Lavoisier/materials of institute Lavoisier
MOF	Metal Organic Framework
NC	Nitrogen-doped Carbon
NP	Nano Particle
ODZ	Ornidazole
PDS	Peroxydisulfate
PMS	Peroxymonosulfate
SEM	Scanning Electron Microscope
SSA	Specific Surface Area
TG	Thermogravimeitry
TEM	Transmission Electron Microscope
TC	Tetracycline
XRD	X-Ray Diffraction
ZCCN	Cobalt metal-organic framework-derived Co-doped porous carbon nitride
ZIFs	Zeolitic Imidazolate Frameworks
