# Supplementary material for: Advantages of Bimetallic Organic Frameworks in the Adsorption, Catalysis and Detection for Water Contaminants
Source: Nanomaterials (Basel). 2023 Jul 28;13(15):2194. doi: 10.3390/nano13152194 (PMC10421224; doi:10.3390/nano13152194)
Supplement: Supplementary file 1 [file nanomaterials-13-02194-s001.zip › nanomaterials-2474576-supplementary.pdf]

Supporting Information

for

Advantages of bimetallic organic frameworks in the adsorption, catalysis and detection for water contaminants

Jun Luo[a], Xiao Luo[a], Yonghai Gan[a], Xiaoming Xu[b], Bin Xu[a], Zhuang Liu[a], Chengcheng Ding[a, *], Yibin Cui[a, *] and Cheng Sun[a, c, *]

[a] *Nanjing Institute of Environmental Sciences, Ministry of Ecology and Environment of the People's Republic of China, Nanjing 210042, PR China*

[b] *Department of Chemistry, Tsinghua University, Beijing, 100084, PR China*

[c] *State Key Laboratory of Pollution Control and Resource Reuse, School of the Environment, Nanjing University, Nanjing 210023, PR China*

* Corresponding author.

E-mail addresses: dcc@nies.org (C. Ding), cyb@nies.org (Y. Cui), envidean@nju.edu.cn, philasun@126.com (C. Sun)

SI. Figures

Figure S1. (a) Schematic of the synthesis mechanism of bimetallic CoNi-MOFs. Reproduced with permission from ref. [1]. Copyright 2021, Elsevier. (b) Schematic drawing of the NiZn-MOFs nanostructure. Reproduced with permission from ref. [2]. Copyright 2022, Elsevier.

Figure S2. (a) Diagrammatic synthesis of CoMo-MI-T. Reproduced with permission from ref. [3]. Copyright 2021, Elsevier. (b) CoNi@C is synthesized via a one-pot solvothermal and pyrolysis reaction. Reproduced with permission from ref. [4]. Copyright 2022, Elsevier. (c) Preparation procedure of CoCe-MOF. Reproduced with permission from ref. [5]. Copyright 2022, Elsevier.

Figure S3. The schematic diagram of the preparation process for $CoFe_2O_4$ NC and the catalytic degradation of BPA by activating PMS and (b) removal efficiency of BPA in different reaction systems within 60 min. Reproduced with permission from ref. [6]. Copyright 2018, Elsevier. Effect of (c) Nb/Co ratio (catalyst = 0.01 g; TC = 100 mL, 0.02 g/L; PMS = 0.3 g/L; pH = 6.02; temperature = 25 °C; the dotted line represents the instability of Co-MOF), (d) Cl^-, (e) NO_3^-, (f) HCO_3^- and (g) $C_2O_4^{2-}$ on TC removal by NbCo-MOF (Nb:Co=1:4). Reproduced with permission from ref. [7]. Copyright 2022, Elsevier.

Figure S4. (a) UV–vis DRS spectra and (b) the band gap energy of as-prepared samples. Reproduced with permission from ref. [8]. Copyright 2022, Elsevier. (c) The process of preparation for ZnTi-MOF and photocatalytic degradation of basic Rhodamine. Reproduced with permission from ref. [9]. Copyright 2021, Elsevier.

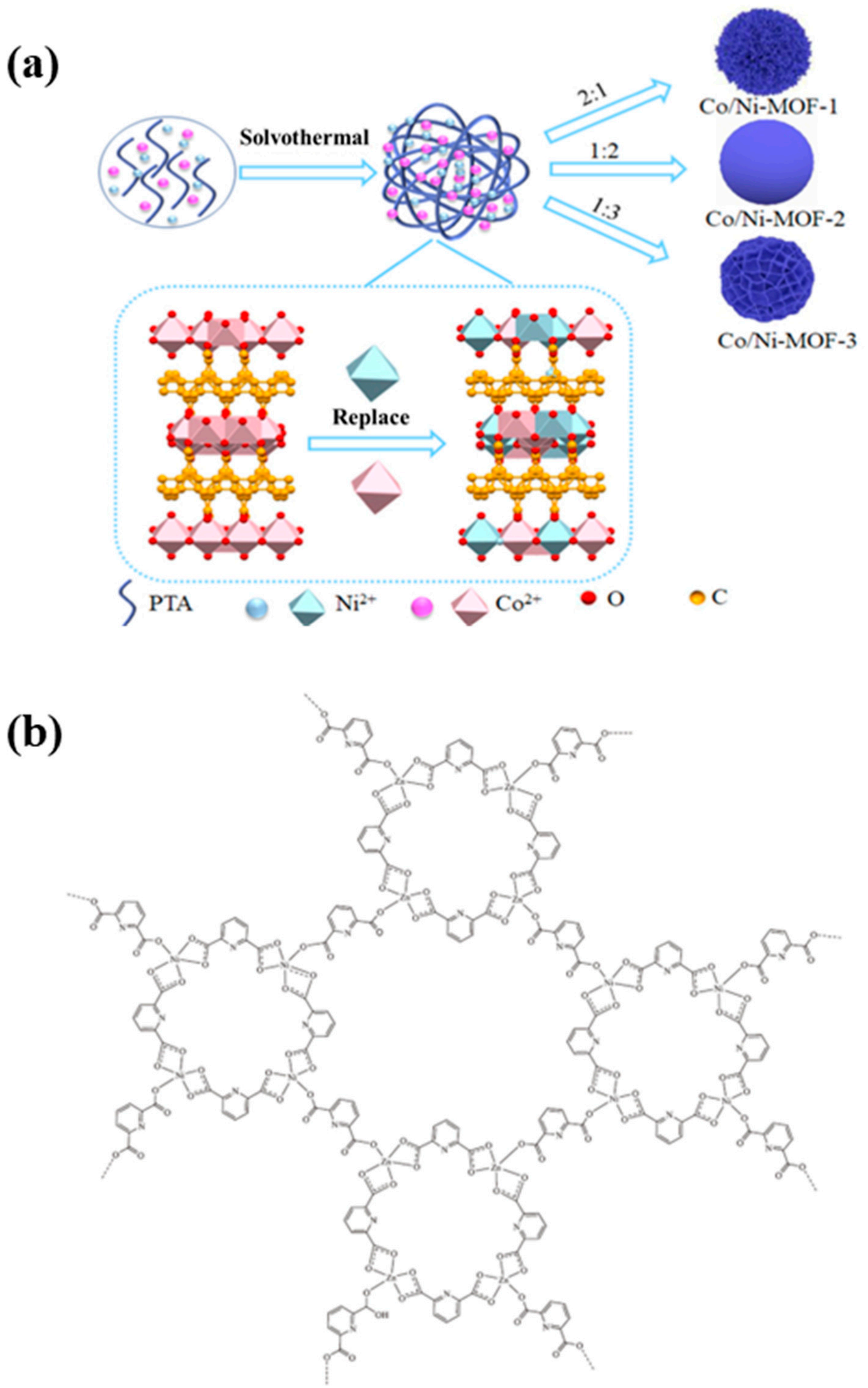

Figure S1. (a) Schematic of the synthesis mechanism of bimetallic CoNi-MOFs. Reproduced with permission from ref. [1]. Copyright 2021, Elsevier. (b) Schematic drawing of the NiZn-MOFs nanostructure. Reproduced with permission from ref. [2]. Copyright 2022, Elsevier.

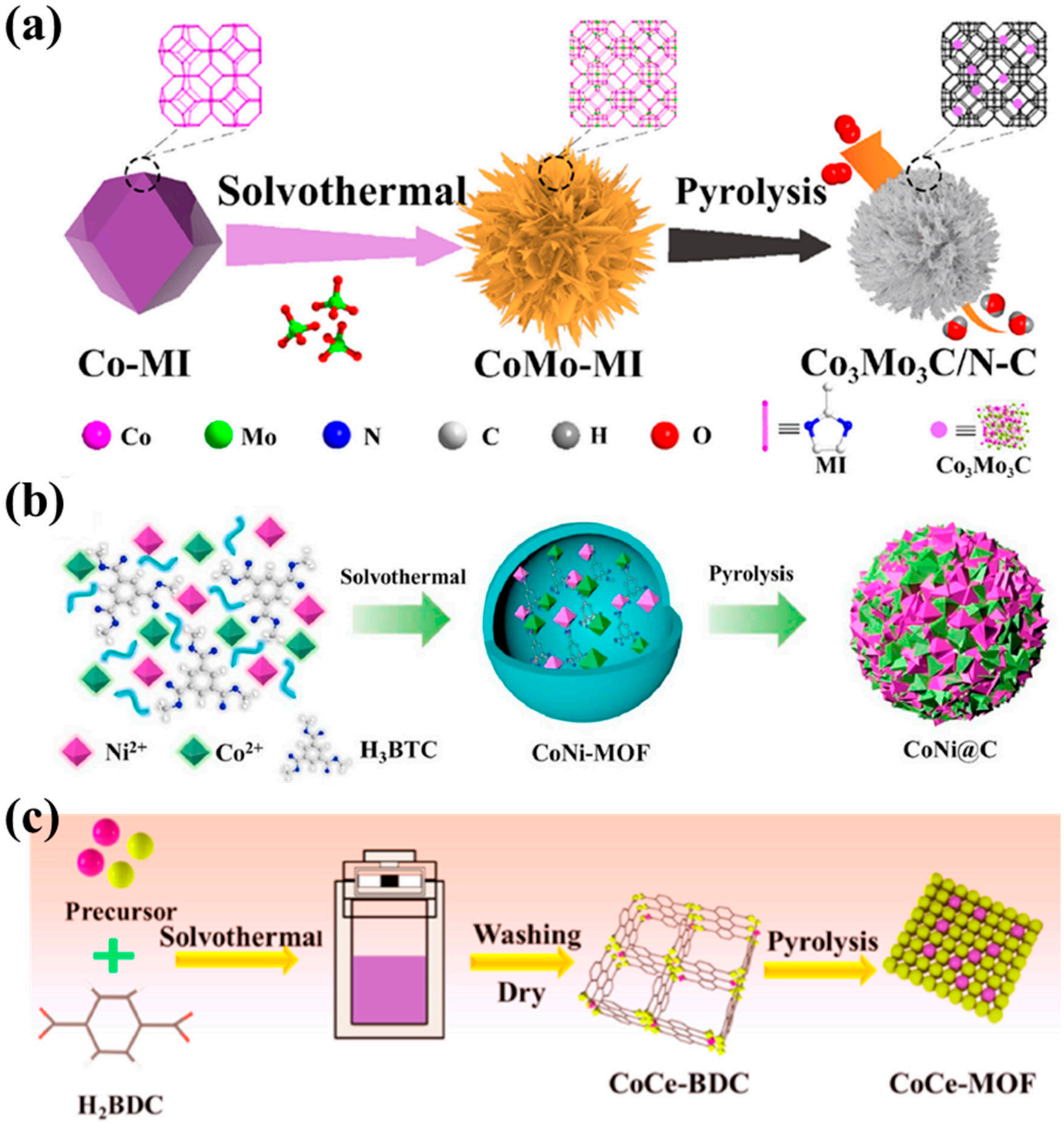

Figure S2. (a) Diagrammatic synthesis of CoMo-MI-T. Reproduced with permission from ref. [3]. Copyright 2021, Elsevier. (b) CoNi@C is synthesized via a one-pot solvothermal and pyrolysis reaction. Reproduced with permission from ref. [4]. Copyright 2022, Elsevier. (c) Preparation procedure of CoCe-MOF. Reproduced with permission from ref. [5]. Copyright 2022, Elsevier.

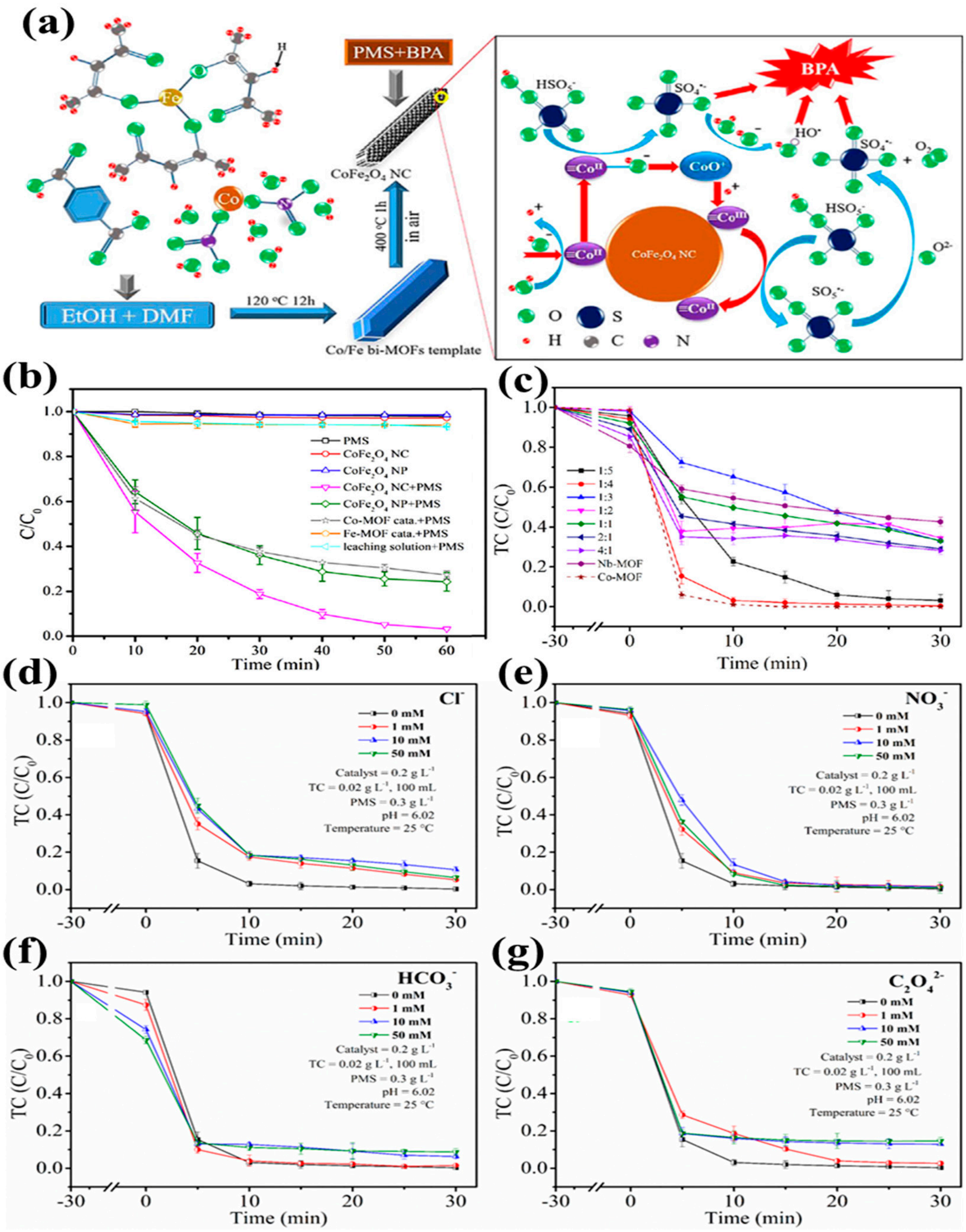

Figure S3. The schematic diagram of the preparation process for $CoFe_2O_4$ NC and the catalytic degradation of BPA by activating PMS and (b) removal efficiency of BPA in different reaction systems within 60 min. Reproduced with permission from ref. [6]. Copyright 2018, Elsevier. Effect of (c) Nb/Co ratio (catalyst = 0.01 g; TC = 100 mL, 0.02 g/L; PMS = 0.3 g/L; pH = 6.02; temperature = 25 °C; the dotted line represents the instability of Co-MOF), (d) Cl^{-}, (e) NO_3^{-}, (f) HCO_3^{-} and (g) $C_2O_4^{2-}$ on TC removal by NbCo-MOF (Nb:Co=1:4). Reproduced with permission from ref. [7]. Copyright 2022, Elsevier.

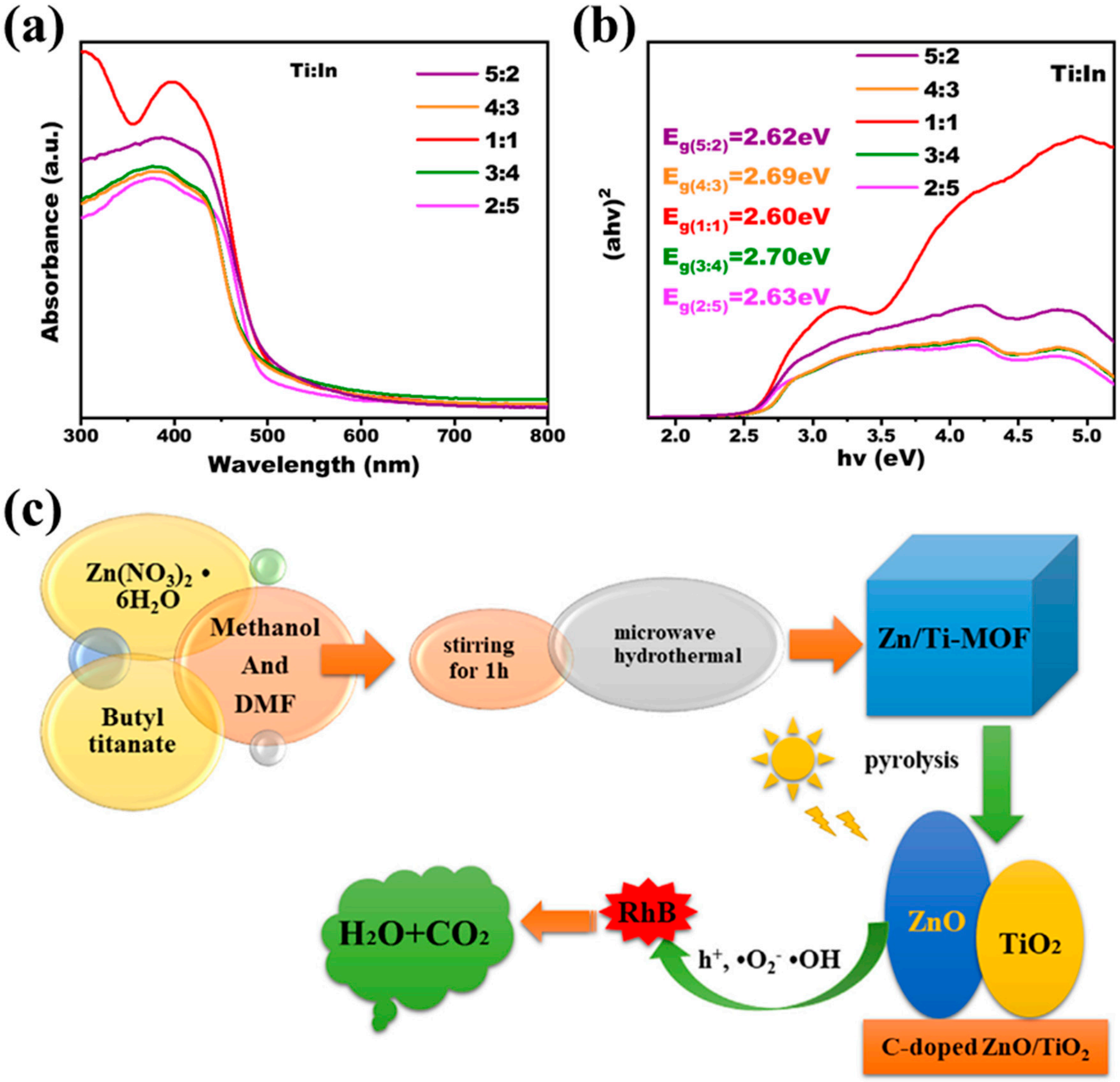

Figure S4. (a) UV–vis DRS spectra and (b) the band gap energy of as-prepared samples. Reproduced with permission from ref. [8]. Copyright 2022, Elsevier. (c) The process of preparation for ZnTi-MOF and photocatalytic degradation of basic Rhodamine. Reproduced with permission from ref. [9]. Copyright 2021, Elsevier.

References

[1] H. Chen, Y. Huo, K. Cai, Y. Teng, Controllable preparation and capacitance performance of bimetal Co/Ni-MOF, Synthetic Met., 276 (2021) 116761. https://doi.org/10.1016/j.synthmet.2021.116761.
[2] W. Suksatan, P. Kazemzadeh, D. Afzali, M. Moghaddam-manesh, N.P.S. Chauhan, G. Sargazi, A controllable study on ultrasound assisted synthesis of a novel Ni/Zn based hybrid MOF nanostructures for *Dextranase* immobilization, Inorg. Chem. Commun., 139 (2022) 109410. https://doi.org/10.1016/j.inoche.2022.109410.
[3] Y. Guo, Q. Huang, J. Ding, L. Zhong, T.-T. Li, J. Pan, Y. Hu, J. Qian, S. Huang, CoMo carbide/nitride from bimetallic MOF precursors for enhanced OER performance, Int. J. Hydrogen Energy, 46 (2021) 22268-22276. https://doi.org/10.1016/j.ijhydene.2021.04.084.
[4] C. Liang, J. He, Y. Zhang, W. Zhang, C. Liu, X. Ma, Y. Liu, J. Gu, MOF-derived CoNi@C-silver nanowires/cellulose nanofiber composite papers with excellent thermal management capability for outstanding electromagnetic interference shielding, Compos. Sci. Technol., 224 (2022) 109445. https://doi.org/10.1016/j.compscitech.2022.109445.
[5] M. Wang, F. Li, J. Dong, X. Lin, X. Liu, D. Wang, W. Cai, MOF-derived $CoCeO_x$ nanocomposite catalyst with enhanced anti-coking property for ethanol reforming with CO_2, J. Environ. Chem. Eng., 10 (2022) 107892. https://doi.org/10.1016/j.jece.2022.107892.
[6] S. Yang, X. Qiu, P. Jin, M. Dzakpasu, X.C. Wang, Q. Zhang, L. Zhang, L. Yang, D. Ding, W. Wang, K. Wu, MOF-templated synthesis of $CoFe_2O_4$ nanocrystals and its coupling with peroxymonosulfate for degradation of bisphenol A, Chem. Eng. J., 353 (2018) 329-339. https://doi.org/10.1016/j.cej.2018.07.105.
[7] Z. Li, S. Ning, H. Zhu, X. Wang, X. Yin, T. Fujita, Y. Wei, Novel NbCo-MOF as an advanced peroxymonosulfate catalyst for organic pollutants removal: growth, performance and mechanism study, Chemosphere, 288 (2022) 132600. https://doi.org/10.1016/j.chemosphere.2021.132600.
[8] M. Li, J. Yuan, G. Wang, L. Yang, J. Shao, H. Li, J. Lu, One-step construction of Ti-In bimetallic MOFs to improve synergistic effect of adsorption and photocatalytic degradation of bisphenol A, Sep. Purif. Technol., 298 (2022) 121658. https://doi.org/10.1016/j.seppur.2022.121658.
[9] Y. Wang, X. Liu, L. Guo, L. Shang, S. Ge, G. Song, N. Naik, Q. Shao, J. Lin, Z. Guo, Metal organic framework-derived C-doped ZnO/TiO_2 nanocomposite catalysts for enhanced photodegradation of Rhodamine B, J. Colloid Interf. Sci., 599 (2021) 566-576. https://doi.org/10.1016/j.jcis.2021.03.167.
